# The association between internet-use-disorder symptoms and loneliness: a systematic review and meta-analysis with a categorical approach

**DOI:** 10.1017/S0033291725000376

**Published:** 2025-03-24

**Authors:** Gemma Mestre-Bach, Ursula Paiva, Leyre San Martín Iniguez, Marta Beranuy, María Martín-Vivar, Nuria Mallorquí-Bagué, Enrique Normand, María Contreras Chicote, Marc N. Potenza, Gonzalo Arrondo

**Affiliations:** 1 Instituto de Investigación, Transferencia e Innovación, Universidad Internacional de La Rioja, La Rioja, Spain; 2 Mind-Brain Group, Institute for Culture and Society (ICS), University of Navarra, Pamplona, Spain; 3 Faculty of Education and Psychology, University of Navarra, Pamplona, Spain; 4 Faculty of Health Sciences, Public University of Navarra, Pamplona, Spain; 5 Grupo de investigación en ciberpsicología. Universidad Internacional de La Rioja; 6 Facultad de Educación y Psicología, Universidad Francisco de Vitoria, Madrid, Spain; 7 Department of Psychology, University of Girona, Girona, Spain; 8 Unidad de Sexología Clínica y Salud Sexual, Consulta Dr. Carlos Chiclana, Madrid, Spain; 9 Doctorat en Medicina i Recerca Translacional, Universitat de Barcelona; 10 Department of Psychiatry, Yale School of Medicine, New Haven, CT, USA; 11 Connecticut Mental Health Center, New Haven, CT, USA; 12 Connecticut Council on Problem Gambling, Wethersfield, CT, USA; 13 Wu Tsai Institute, Yale University, New Haven, CT, USA; 14 Yale Child Study Center, Yale School of Medicine, New Haven, CT, USA; 15 Department of Neuroscience, Yale University, New Haven, CT, USA

**Keywords:** addictive behaviors, internet addiction, internet gaming disorder, internet-related disorders, loneliness, online gambling disorder, problematic smartphone use

## Abstract

Loneliness may lead individuals to spend more time on the internet and increase the likelihood of experiencing internet-use disorders. Similarly, individuals with internet-use disorders may feel lonelier. In the present systematic review and meta-analysis, pre-registered in PROSPERO (CRD42023390483), we quantified associations between internet-use-disorder symptoms (e.g. internet gaming disorder and online gambling disorder) and loneliness. We searched PubMed, Web of Science, and an institutional database aggregator for references that compared degrees of loneliness in groups of individuals with and without symptoms of internet-use disorder. Means and standard deviations of loneliness, or alternatively, odds ratios, were transformed into Cohen’s d for statistical pooling through a random-effects model. After screening 2,369 reports, we extracted data from 23 studies. The total number of individuals across the studies was 36,484. Participants were between 13 and 30 years of age (median 20). The pooled difference between those with and without internet-use-disorder symptoms yielded a standardized effect (Cohen’s d) of 0.53 (95% CI 0.35–0.7). While heterogeneity was high, there was no indication of publication or small sample biases. Similar effect sizes were found when limiting to specific types of internet-use disorder symptoms. Moreover, meta-regressions did not show an effect of age, sex, or sample size. Individuals with symptoms of internet-use disorders scored 49.35 (43.84–54.85) points on the UCLA-Loneliness scale on average, compared to 43.78 (37.47–50.08) in individuals without symptoms of internet-use disorders (Standardized Mean Difference: 5.18, 95% CI = 2.05–8.34). Individuals with internet-use-disorder symptoms experience greater loneliness. The effect appears moderately sized.

## Introduction

### Internet-use disorders

Since its introduction in the 1980s, the internet has evolved into a global phenomenon. The widespread use of the internet has been linked to the emergence of specific internet behaviors including gambling, video gaming, buying/shopping, pornography viewing and other sexual behaviors, and social networking, among others (Ioannidis et al., 2016). In some cases, these behaviors may develop into internet-use disorders, which are increasingly recognized, affect all age groups, and have become a challenge for mental health (Fineberg et al., 2018, 2022). Internet-use disorders are characterized by functional impairment resulting from excessive engagement in internet behaviors, marked by addictive, impulsive, or compulsive elements (Ioannidis et al., 2016; Király, Griffiths, & Demetrovics, 2015). Some ([online] gambling disorder (GD), [internet] gaming disorder (IGD), and [online] compulsive sexual behavior disorder (CSBD)/problematic pornography use (PPU)) but not all ([online] compulsive buying/shopping disorder (CBSD), social network use disorder) putative internet-use disorders have been recognized as formal mental disorders in either of the two major diagnostic manuals, the Diagnostic and Statistical Manual of Mental Disorders (DSM-5) or the International Classification of Diseases (ICD-11), and some (GD, IGD) have been classified as behavioral addictions (Banz, Yip, Yau, & Potenza, 2016; Brand et al., 2025; Dell’Osso et al., 2021; Yau & Potenza, 2015). In this manuscript, we use the term symptoms of internet-use disorders, as many types of internet-use behaviors (e.g. use of social media) do not have specific formal clinical diagnoses, even though the diagnosis of “other specified disorder due to addictive behaviors” has been proposed for use for such entities as this is a formal clinical diagnosis in the ICD-11.

### Loneliness

Loneliness is generally understood as an inherently subjective experience, where individuals perceive a gap between their desired or expected levels of social interaction and their actual experiences (Baumeister & Leary, 1995). Loneliness has been linked to distressing emotions that arise from feeling that one’s social needs are not being met, both in terms of the number and the quality of social interactions. Some theories distinguish between: (a) emotional loneliness, when there is no close attachment figure, and (b) social loneliness arises when there is an absence of a wider social network, leading to a reduced sense of belonging to a group or community (Yanguas, Pinazo-Henandis, & Tarazona-Santabalbina, 2018).

### Relationships between loneliness and internet-use disorders

It is important to highlight that internet use and loneliness can be part of normal behavior and subjective experience, but they can also reach clinically significant or problematic levels. Hence, they may be conceptualized as categorical and dimensional constructs. Additionally, both internet use and loneliness problems may relate to psychopathological concerns (i.e. they may operate trans-diagnostically and co-occur with other issues such as social anxiety, dysthymia, emotional dysregulation, and poor social skills).

The possible association between loneliness and internet use disorders has been explored in recent years, and different hypotheses and theories have emerged. On the one hand, the displacement hypothesis suggests that the time an individual spends engaging in internet behaviors displaces time that could be spent engaging offline in quality social interactions, potentially leading to negative consequences such as loneliness (Kraut et al., 1998). On the other hand, loneliness has been identified as one of the main predisposing variables of internet-use disorders in the Interaction of Person-Affect-Cognition-Execution (I-PACE) model, which has focused on biopsychosocial factors involved in the development and maintenance of these disorders (Brand et al., 2019; Brand, Young, Laier, Wölfling, & Potenza, 2016). Therefore, lonely individuals could develop internet-use disorders based on using the internet to regulate negative emotional states; that is, as a maladaptive coping strategy (Moretta & Buodo, 2020). In this line, the problem behavior theory has also been proposed (Jessor, 1987). This theory suggests that the emergence of problem behaviors, in this case internet-use disorders, may reflect efforts to address needs, such as social ones, that have been unmet. Loneliness would thus be a consequence of an unmet social need that would lead the individual to develop online problem behaviors, which could perpetuate the cycle of loneliness (Wang & Zeng, 2024). Therefore, the relationship between loneliness and internet-use disorders may be complex and bidirectional.

Previous reviews have primarily focused on specific internet-use disorders, such as general problematic internet use (Zhang, Li, Zhang, B., & Jia, 2024), smartphone addiction (Ge et al., 2023), or IGD (Zhuang et al., 2023). However, these reviews often lack a defined protocol, limiting their methodological consistency. Additionally, they are constrained by specific characteristics, such as focusing exclusively on high-school students (Ge et al., 2023), including only longitudinal studies (Zhang et al., 2024), or focusing on loneliness as one of many potential risk factors (Zhuang et al., 2023). However, we aimed to go further and explore relationships with specific internet-use concerns (even those that are not recognized in current diagnostic taxonomies, such as problematic use of social media) as their diagnostic recognition may not be related to their putative relationship with loneliness, and exploring only generalized internet addiction does not account for potential differences among specific types of internet-use disorders. Therefore, the primary aim of the present systematic review and meta-analysis was to analyze the possible association between loneliness and symptoms of specific internet-use disorders. Two research questions addressed by this systematic review and meta-analysis: (1) Whether individuals with internet-use-disorder symptoms experienced higher levels of loneliness compared to those without these symptoms; and (2) Whether there were differences in the levels of loneliness according to the type of internet-use disorder.

## Methods

### Protocol and registration

This systematic review and meta-analysis followed the Preferred Reporting Items for Systematic Reviews and Meta-Analyses (PRISMA) guidelines (Page et al., 2021). It was registered in the International Prospective Register of Systematic Reviews of the National Institute for Health Research (PROSPERO; registration number: CRD42023390483; registration date: 7 February 2023; registration website: https://www.crd.york.ac.uk/prospero/display_record.php?RecordID=390483).

### Eligibility criteria

#### Study characteristics

The inclusion criteria were:

- Population:
  - human beings,
  - community and clinical samples,
  - any sexual orientation,
  - any sex,
  - any age,
  - any country.
- Assessment:
  - articles that included a standardized assessment of loneliness. While we expected loneliness to be typically defined as a continuous variable derived from answering a questionnaire (i.e. dimensional characterization of loneliness), categorical characterizations of loneliness derived from setting a threshold in validated scales were also accepted (i.e. dividing the sample into lonely and non-lonely individuals);
  - articles that assessed one or more internet-use disorders, in which a diagnosis was obtained through clinical diagnostic criteria, a score was obtained over the threshold in a psychometric instrument assessing these clinical conditions, via a self-reported internet-use disorder, or through the result of an algorithmic identification of cases in administrative registers. Definitions of internet-use disorders accepted in the DSM-5 or ICD-11 or previous versions were prioritized in the search through the terms included (e.g. IGD, online GD, online CBSD, and online CSBD/PPU). However, it was also decided to include other problematic forms of internet use not covered in these manuals, following the definitions provided by the authors of the primary references. Hence, we also accepted studies if authors used a validated questionnaire to measure misuse of the internet or addiction to internet use in general (such as the internet addiction scale) or specific facets or activities related to it (such as problematic use of social media or smartphones) (Brand et al., 2022; Fineberg et al., 2022). In the protocol, we specified to include both categorical and dimensional categorizations of internet-use disorders, but we realized in the screening phase that this was not ideal, due to the high number of potential inclusions. Hence, we decided to limit our inclusion criteria to studies where internet-use disorders were defined categorically.
- Articles specifically evaluating the association between loneliness and internet-use-disorder symptoms. Similarly, although we initially planned to include any behavioral addiction, most of the references meeting our criteria focused on internet-use disorders. Therefore, we adjusted our criteria to only include these studies.

The exclusion criterion was the assessment of loneliness and/or internet-use disorders through items designed specifically for the study or drawn from validated scales in an ad-hoc manner (i.e. without a proper validation analysis). Short versions of existing scales (such as the UCLA-LS) were accepted. Studies validating a novel scale or version of either loneliness or internet-use disorder symptoms assessments were accepted.

#### Report characteristics

We included the following: (1) peer-reviewed articles, (2) written in English or Spanish, (3) published until 11th of March 2024, (4) with any design, and (5) having a quantitative methodology.

We excluded: gray literature, books, chapters, conference papers/abstracts, case reports or case series, meta-analyses, and systematic reviews. Articles without an abstract and publications that were not full articles were excluded.

### Information sources and search strategy

PubMed-Medline and Web of Science databases were searched during January 2023. Additionally, we also searched UNIKA, an institutional database aggregator based on the EBSCO-host system that combines items from over a hundred databases (see Supplementary Table S1). The full search query and the rationale for its construction are included in Supplementary Table S2. The search was updated on the 11th of March 2024.

### Study selection

Records were imported into Covidence (Veritas Health Innovation, 2024), where duplicates were automatically and manually identified. Screening, full-text selection, and data extraction were done independently in pairs by members of the research team after piloting the full process by the study senior authors. Consensus was reached in cases of disagreements, with additional consultation with senior authors when indicated.

### Data collection process and data items

The data extracted from included studies were: first author and year, country, study design, setting, type of internet-use disorder, assessment of internet-use disorders, assessment of loneliness, sample size (N), sex (percentage of females), and age in years. Information on the date and origin of the data, and socioeconomic, demographic, race/ethnicity characteristics of the samples, as well as comorbidities, were also obtained. Key data on the included studies and their samples and statistics were extracted to a shared datasheet in Google Drive.

### Study risk of bias assessment

We planned to evaluate the risk of bias using the Newcastle-Ottawa scale (Wells et al., 2000), employing the version for cross-sectional studies (Herzog et al., 2013). However, we needed to modify it to make it applicable to our pool of studies. Since we expected loneliness to be evaluated solely through questionnaires, the question related to the assessment of the outcome was eliminated (Table S3).

### Statistical analyses

Statistical analyses were conducted in Stata 18 (StataCorp, 2023). In each analysis, a single Cohen’s d, representing the difference in loneliness between the sample of individuals with and without internet-use-disorder symptoms, was generated for each study. Cohen’s d was typically calculated using the mean and standard deviation of loneliness in the two groups. Whenever loneliness was reported as a categorical variable, we transformed the odds ratios (or calculated them from raw data if needed) into Cohen’s d values (Borenstein, Hedges, Higgins, & Rothstein, 2009). Other instances in which we performed additional transformations occurred when there were more than two groups for which loneliness was reported. For example, studies frequently reported three categories of internet-use disorders: no internet-use disorder, at-risk internet-use disorder, and internet-use disorder. In these and other similar cases, means and standard deviations were combined across groups to obtain a single value using a fixed-effects model (Borenstein et al., 2009), as they referred to independent groups of individuals. In the specific case named, a higher threshold of symptoms for internet-use disorder was used, so the at-risk group was combined with the no internet-use-disorder group to make results more comparable across studies. The effect of this decision was also evaluated through a sensitivity analysis. Once we had a single value per study for each analysis, a random-effects model with a restricted maximum likelihood estimation of parameters was used to pool data across studies (Cochrane Methods, 2024), as recommended by the Cochrane collaboration. In cases where only graphical data were presented in a study, we used the web tool WebPlotDigitizer to obtain numerical values of loneliness (Rohatgi, 2024). We also calculated the pooled prevalence for specific groups of internetuse disorders. Studies were combined into five subcategories: (1) general internet-use addiction when evaluated with the IAS or other similar measures that aimed to evaluate all aspects of dysfunctional use of the internet; (2) IGD, (3) specific internet facets, which included studies evaluating specific problematic behaviors conducted via the internet such as Facebook addiction, problematic video streaming, problematic use of social media and online gambling, (4) smartphone addiction, for studies centered specifically in the misuse of this specific device, (5) mixed conditions, for studies that evaluated more than one of the previous categories. While IGD could be classified as a specific facet of internet use, it was treated separately because it is currently recognized as a disorder in the DSM-5.

For sensitivity analyses, we evaluated the effect of using a less stringent threshold of internet-use disorders when possible, and we also conducted a leave-one-out meta-analysis to study the effect of specific studies on the overall pooled effect. We included the mean age, the percentage of women, and the world region in meta-regression analyses. Finally, we pooled the mean and standard deviations for individuals with and without internet-use disorder symptoms of the studies using the 20-item version of the University of California Loneliness Scale (UCLA-LS) (Russell, 1996), and the standardized mean difference was also obtained. Small-sample bias was assessed using funnel plots and Egger’s tests, while heterogeneity was evaluated with i squared.

### Ethics

N/A

## Results

### Study selection

**Figure 1.**
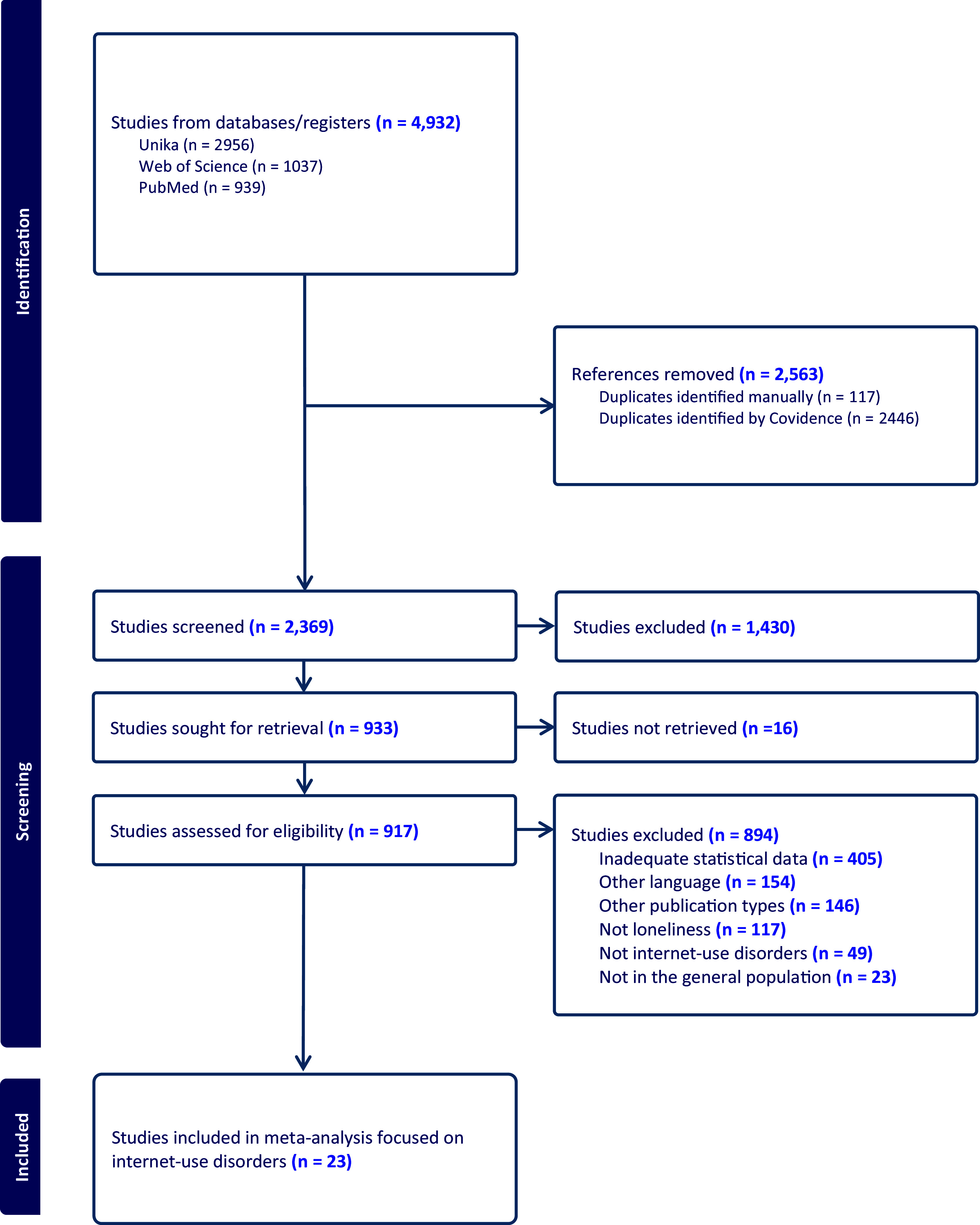
PRISMA flow diagram.

The median Cohen’s Kappa agreement between reviewer pairs for the screening phase was 0.48, whereas the kappa agreement between reviewers reached 0.72 during the full-text evaluation. The flowchart for the bibliographic search and extraction is found in Figure 1. Additional details are found in the Supplementary Materials and Table S4.

Twenty-three studies were finally included in our data synthesis (Akbari et al., 2023; Aktepe, Olgaç-Dündar, Soyöz, & Sönmez, 2013; Erol & Cirak, 2019; Hardie & Tee, 2007; Hou et al., 2019; Jeon, Jeong, Lee, & Kim, 2022; Karaibrahimoglu et al., 2023; King, Delfabbro, Zwaans, & Kaptsis, 2013; Lin & Chiao, 2024; Liu, Yu, Kong, & Zhou, 2023; Myrseth, Olsen, Strand, & Borud, 2017; Sangram & Gawas, 2020; Orsolini, Longo, & Volpe, 2023; Paschke, Napp, & Thomasius, 2022; Reed, Vile, Osborne, Romano, & Truzoli, 2015; Shettar, Karkal, Kakunje, Mendonsa, & Chandran, 2017; Shi, Wang, & Zou, 2017; Smith & Short, 2022; Van Rooij et al., 2014; Verma, Khan, Singh, & Saxena, 2023; A. Wang, Wang, Zhu, & Shi, 2022; Yu et al., 2022; Zakaria et al., 2023).

### Study characteristics

#### Overview of studies and samples

The main characteristics of the studies are included in Table 1. Most studies had been published in Asia (4 in the Middle East, 11 in other parts of Asia), 5 in Europe, 2 in Oceania, and 1 in the Caribbean. All studies, except three, had a cross-sectional design (with these three having a cohort or longitudinal design), and all used community samples.

**Table 1.** Description of included studies

References	Country	Study design	Setting	Type of internet-use disorder symptom	Assessment of internet-use disorder symptom	Assessment of loneliness	N	Sex (% females)	Age in years
Akbari et al. (2023)	Iran	Cross-sectional	Community	Multiple internet facets (problematic social media use &gambling & gaming)	IGDT–10, PGSI, BSMAS	UCLA-LS-SF(3)	2,390	65.06	16.01 (sd=/1.38)
Aktepe et al. (2013)	Turkey	Cross-sectional	Community	General internet use	IAS	UCLA-LS-SF(4)	1,645	42.6	16.32 (sd = 1.08)
Erol & Cirak (2019)	Turkey	Cross-sectional	Community	General internet use	IAS	UCLA-LS	489	56.9	20.54 (sd = 1.6)
Hardie & Tee (2007)	Australia	Cross-sectional	Community	General internet use	IAS	Wittenberg’s Emotional and Social Loneliness Scale	96	51.04	26.9 (sd = 9.28)
Hou et al. (2019)	China	Cross-sectional	Community	General internet use	IAS	UCLA-LS	64	0	20.39 (sd = 1.44)
Jeon, Jeong, Lee, & Kim (2022)	Korea	Cohort/longitudinal	Community	Internet gaming	IAS	UCLA-LS-SF(10)	778	51	13.5 (r:10–17)
Karaibrahimoglu et al. (2023)	Turkey	Cross-sectional	Community	Specific internet facet (online gambling)	OGAS	Social and emotional loneliness scale for adults SELSA-S	449	52.78	r:18–32
King et al. (2013)	Australia	Cross-sectional	Community	Multiple internet facets (general internet use & internet gaming)	PTU	UCLA-LS	1,214	49.58	14.8 (sd = 1.5)
Lin & Chiao (2024)	Taiwan	Cohort/longitudinal	Community	General internet use	Chen’s Internet Addiction Scale	De Jong Gierveld Short Scale	1,885	45.89	30.3
Liu et al. (2023)	China	Cross-sectional	Community	Problematic smartphone use	SAS-C	UCLA-LS	221	97.29	25.26 (sd = 3.16)
Myrseth et al. (2017)	Norway	Cross-sectional	Community	Internet gaming	GAS	UCLA-LS-SF(8)	1,017	19.7	19.5 (sd = 1)
Orsolini et al. (2023)	Italy	Cross-sectional	Community	General internet use	IAS	The Italian Loneliness Scale	1,643	68.7	21.8 (sd = 1.7)
Paschke et al., 2022	Germany	Cross-sectional	Community	Specific internet facet (problematic video streaming)	STREDIS-A	UCLA-LS-SF(6)	959	46.92	13.5 (sd = 2.16)
Reed et al. (2015)	UK	Case–control	Community	General internet use	IAS	UCLA-LS	505	52.47	29.73 (sd = 13.65)
Sangram (2020)	India	Cross-sectional	Community	General internet use	IAS	UCLA-LS	200	50	18.9 (r:17–20)
Shettar et al. (2017)	India	Cross-sectional	Community	Specific internet facet (Facebook addiction)	BFAS	UCLA-LS	100	46	27.55 (sd = 2.88)
Shi et al. (2017)	China	Cross-sectional	Community	General internet use	IADQ	Asher’s Child Loneliness Scale	3,289	49.83	15.77
Smith & Short (2022)	Trinidad and Tobago	Cross-sectional	Community	Specific internet facet (problematic social media use)	Problematic TikTok Use Scale	UCLA-LS-SF(3)	173	67.3	23.61 (sd = 5.82)
Van Rooij et al. (2014)	Netherlands	Cross-sectional	Community	Internet gaming	VAST	UCLA-LS-SF(10)	8,478	51	14.2 (sd = 1.1)
Verma et al. (2023)	India	Cross-sectional	Community	Problematic smartphone use	SAS-SV	UCLA-LS-SF(6)	402	52.5	22.4 (sd = 2.2)
Wang et al. (2022)	China	Cohort/longitudinal	Community	Problematic smartphone use	SAS-SV	UCLA-LS-SF(8)	7,434	48.2	19.67 (sd = 1.15)
Yu et al. (2022)	China	Cross-sectional	Community	Internet gaming	DSM–5 IGD Symptom checklist	UCLA-LS-SF(3)	2,573	48	13.1 (sd = 1.3)
Zakaria et al. (2023)	Malaysia	Case–control	Community	General internet use	IAS	UCLA-LS	480	73.1	21.01 (sd = 1.29)

*Note*: Cross-sectional refers to studies in which loneliness and internet-use-disorder symptoms are evaluated in groups of individuals that have not been selected due to having internet-use-disorder symptoms or loneliness. A design in which a group of individuals with and without loneliness or a group of individuals with and without internet-use disorders were recruited was also accepted, but no studies were found in this regard.

BFAS, Bergen Facebook Addiction Scale; BSMAS, Bergen Social Media Addiction Scale; DSM-5, Diagnostic and Statistical Manual of Mental Disorders; GAS, Gaming Addiction Scale; IADQ, Internet Addiction Diagnostic Questionnaire; IAS, Internet Addiction Scale; IGD, Internet Gaming Disorder; IGDT-10, Internet Gaming Disorder Test-10; OGAS, Online Gambling Addiction Scale; PGSI, Problem Gambling Severity Index; PTU, Pathological Technology Use Checklist; SAS-C, Smartphone Addiction Scale for College Students; SAS-SV, Smartphone addiction scale short version; STREDIS-A, Streaming Disorder Scale for Adolescents; VAST, Video Game Addiction Test; UCLA-LS, University of California, Los Angeles Loneliness Scale; UCLA-LS-SF, University of California, Los Angeles Loneliness Scale – Short Form (with number of items included in brackets).

The total number of individuals across studies was 36,484, with a median sample size of 778 (range 64–8478). Studies, for the most part, involved young individuals, with a median mean age of 20.03 years (range of means: 13.1–30.3) and had a similar number of men and women (median percentage of women 51%, range 0%–97.29%). Data detailing the ethnicity or socioeconomic status of the participants was hard to homogenize across studies and was overall scant (Tables S5 to S8).

The most typically studied condition was internet addiction. Ten studies focused on internet addiction in general, whereas three analyzed data on a more specific facet of internet addiction (problematic video streaming, problematic use of social media/Facebook addiction, or online GD). Four research articles focused on IGD and three on problematic smartphone use. Two more pooled together IGD and online GD.

#### Assessment of internet-use-disorder symptoms and loneliness

The Internet Addiction Scale (IAS; Young, 1998) was the most frequently used scale to identify internet-use-disorder symptoms, (9 studies). This 20-item scale assesses compulsive use of the internet, considering compulsivity, escapism, and dependency.

Loneliness was typically quantified using a variant of the University of California, Los Angeles – Loneliness Scale (UCLA-LS; Russell, 1996). This scale assesses direct feelings of loneliness and also asks about the respondent evaluations of different qualitative features of their social networks. Eighteen studies used a version of this scale, with eight studies using the 20-item version and ten using shorter adaptations that had between 3 and 10 items. Both the IAS and UCLA-LS are self-reported measures.

### Meta-analyses findings

The median numbers of participants without and with internet-use-disorder symptoms were 384 (range 32–3434) and 125 (range 8–4892), respectively, with additional details on these samples provided in Supplementary Tables S9 to S12. Effect sizes on the differences between groups are reported in Table S13. Most studies had a high risk of bias, obtaining between 1 and 4 stars (median 2) out of 8 in our modified version of the NOS (Table S14). It should be noted that several questions had reduced variability across studies, hence limiting their capability to discriminate between studies at a high risk of bias versus those at a low risk of bias.

**Figure 2.**
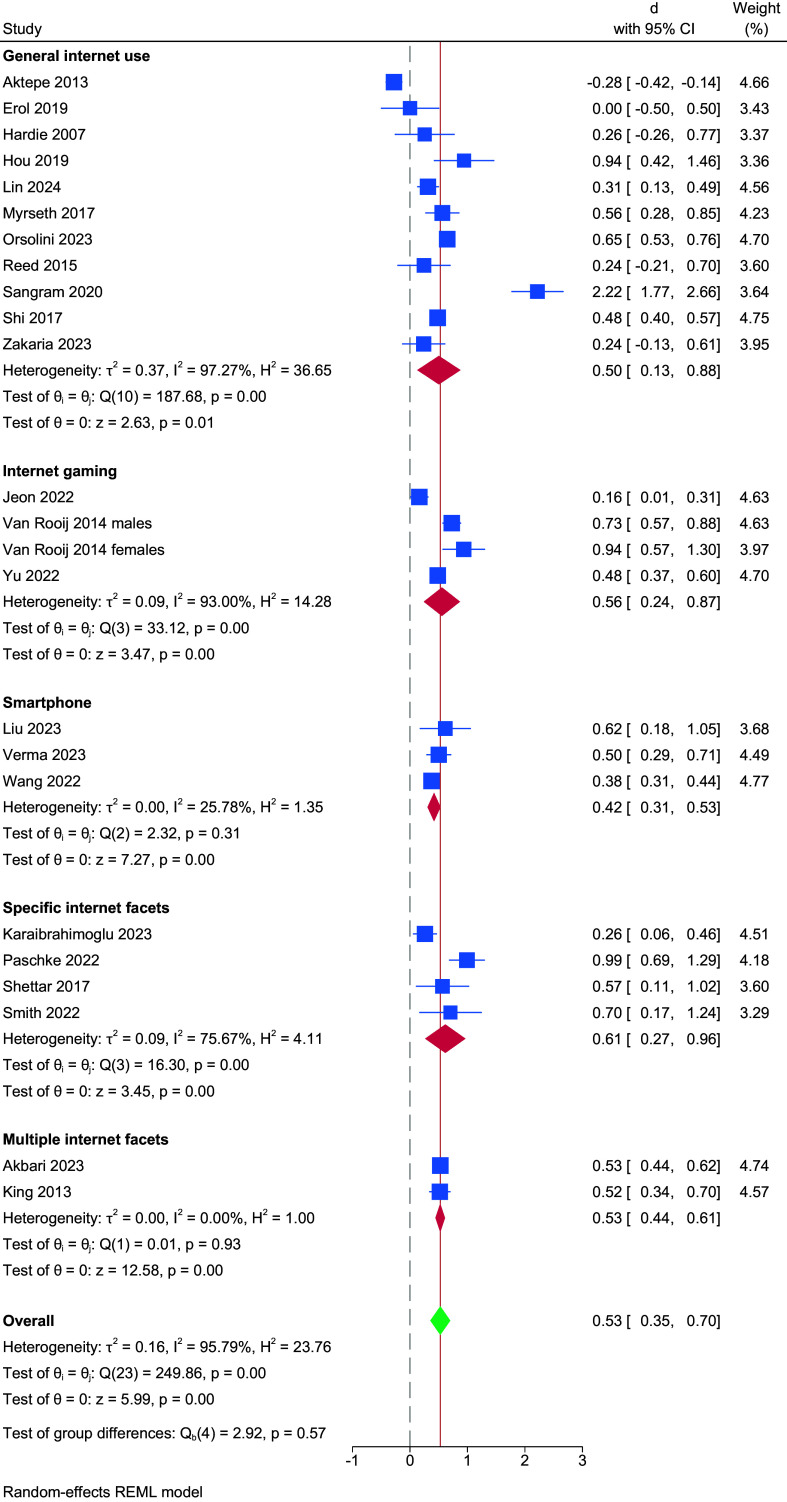
Forest plot. Pooled difference between those with and without internet-use-disorder symptoms. *Note*: The dashed line indicates the point of no effect. The continuous line marks the pooled effect.

The pooled difference between those with and without internet-use-disorder symptoms yielded a standardized effect (Cohen’s d) of 0.53 (95% CI 0.35–0.7), as observed in Figure 2. While heterogeneity was high (i2 = 95.79), there was no indication of publication or small sample biases (p = 0.17) (Figure S1). Effect sizes were similar to the overall pooling when dividing studies according to the type of internet-use-disorder symptomatology assessed. The average effect size for general internet-use disorder (when evaluated with the IAS or similar measure) was 0.5 (CI = 0.13–0.88), 0.56 for IGD (CI = 0.24–0.87), 0.42 for smartphone addiction, 0.61 (CI = 0.27–0.96) for specific internet-use-disorder facets (Facebook addiction, problematic video streaming, problematic use of social media and online gambling, a study for each), and 0.53 (CI = 0.44–0.61) for studies reporting on multiple internet-use-disorder facets in a pooled way (e.g. problematic use of social media, gambling, and gaming).

These results were robust to the elimination of specific studies. The biggest change derived from the elimination of the study by Sangram et al., which reduced the effect size to 0.46 (CI = 0.34–0.58) (Figure S2). Results did not change when we used a lower severity threshold in those studies including multiple levels of risk (i.e. considering those “at risk” as having a disorder) (see Figure S3). Meta-regressions found no effect of age, sex, sample size, or world region (see Table S15). Individuals with internet-use-disorder symptoms obtained 49.35 (43.84–54.85) points in the UCLA-LS on average, compared to 43.78 (37.47–50.08) in individuals without internet-use-disorder symptoms (SMD 5.18, 95% CI = 2.05–8.3), as seen in Figures S4 to S6.

## Discussion

The first research question addressed by this systematic review and meta-analysis was whether individuals with internet-use-disorder symptoms experience higher levels of loneliness compared to those without these concerns. To address this question, 23 studies were included, encompassing a total of 36,484 individuals aged between 13 and 30 years. The findings highlighted that the overall difference between individuals with and without internet-use-disorder symptoms showed a moderate effect. Therefore, those individuals with internet-use-disorder symptoms tended to feel lonelier than those without these symptoms. This moderate effect was observed in studies largely involving community samples. It is possible that the differences in loneliness would be greater if a clinical population with internet-use-disorder symptoms and controls were compared.

There have been several systematic reviews published recently on the association between loneliness and internet-use-disorder symptoms, including four published after the registration of our protocol and systematic search (Ge et al., 2023; Wang & Zeng, 2024; Zhang et al., 2024; Zhuang et al., 2023). However, our study stands out due to several key characteristics. First, we followed a pre-established protocol, ensuring greater transparency, consistency, and reproducibility in our review process. Second, by including a wider variety of conditions when considering problematic use of the internet, we captured a broader range of articles, enabling a more comprehensive analysis. This approach not only facilitated a deeper understanding of the spectrum of disorders/concerns but also allowed for examination of whether differences existed between them, rather than treating internet-use disorders solely as a singular or generic category. Moreover, by using a scoring system above a threshold to define addiction symptomatology, we went beyond the continuous-variable analyses commonly used in previous research. Categorical research, such as our study, can offer unique clinical insights as it highlights differences between individuals with and without a significant concern. Since the field of behavioral addictions, and specifically, internet-use disorders, are arguably at the stage of re-definition, our study and other similar ones that use a categorical approach can inform task forces involved in the modification of current diagnostic manuals, as well as clinicians involved with the treatment of individuals at the extreme of the distribution. It should be noted, however, that dimensional studies and meta-analyses are also of high importance to describe the full range of variability across populations and define norms.

When considering the larger literature, our findings are consistent with those of previous studies focused specifically on internet addiction that have used a dimensional approach. Mozafar Saadati, Mirzaei, Okhovat, and Khodamoradi (2021), in their systematic review and meta-analysis including 26 articles that evaluated a correlation between internet addiction and loneliness, found a moderate positive association (r = 0.15 (95% CI 0.13–0.16)) between both factors. Similarly, Wang and Zeng (2024) reported in their meta-analysis that loneliness was positively correlated with internet addiction (r = 0.291, p < 0.001).

All in all, our meta-analysis supplements previous efforts in this area of research, while being consistent with them in finding a moderate-sized relationship. This is especially important since differences in inclusion and exclusion criteria and search strategies led to a small overlap in the included studies across meta-analysis. Our results provide additional evidence of the importance of loneliness in internet-use disorders.

The three longitudinal studies included in this meta-analysis provide valuable insights into the mechanisms and potential risk factors associated with problematic use of the internet and smartphones. Three-year data from 778 Korean adolescents highlighted the mediating role of self-control and aggression in the relationship between negative effects (e.g. academic stress and loneliness) and IGD (Jeon et al., 2022). Their findings emphasize an important role of academic stress, especially in high-risk groups. Lin and Chiao (2022) explored the long-term effects of adverse childhood experiences (ACEs) on problematic use of the internet in young adults, demonstrating that ACEs indirectly influence problematic use of the internet through hostility and emotional loneliness, underscoring the lasting psychological consequences of childhood adversity. Finally, Wang et al. (2022) conducted a three-wave longitudinal study among 7,434 Chinese college students and identified depressive symptoms, social anxiety, loneliness, family conflicts, and academic stress as significant predictors of problematic smartphone use. Together, these studies suggest that emotional and psychosocial factors, such as loneliness, are key contributors to internet-use conditions and highlight the importance of early interventions targeting these vulnerabilities to prevent negative outcomes. A recent meta-analysis of longitudinal studies (Zhang et al., 2024) points in this same direction, with loneliness being related to internet-used conditions bidirectionally. Therefore, the displacement theory (Kraut et al., 1998), the I-PACE model (Brand et al., 2019, 2016) and the problem behavior theory (Jessor, 1987) remain to be tested further to investigate their role in the etiology of these conditions.

The second research question addressed by this systematic review and meta-analysis was whether there are differences in the levels of loneliness according to the type of internet-use disorder. We considered it important to compare different types of internet-use disorders, as this was one of the aspects suggested for future research by the authors of the I-PACE model (Brand et al., 2019, 2016). However, we found that effect sizes were similar to the overall pooling when dividing studies according to the type of internet-use disorder assessed. Previous meta-analyses have often combined all types of internet-use disorders into a single category. However, Wang and Zeng (2024) studied the moderating effect of measurement tools and did not find a significant result either.

This finding may be attributed to the possibility that the underlying mechanisms involving loneliness are similar across different types of internet-use disorders. Despite each internet-use disorder having its unique characteristics and nature, internet-use disorders may all be linked to reduced social skills and social interactions, which may exacerbate feelings of loneliness (Kim, LaRose, & Peng, 2009). Additionally, individuals with internet-use disorders, regardless of the specific type, might exhibit emotional regulation problems in response to negative emotions, like feelings of loneliness (Brand et al., 2019, 2016). Moreover, loneliness and internet-use disorders may partly share underlying factors, such as dysregulation, reduced social skills, social anxiety, or dysthymia which may contribute to their association. The co-occurrence of internet-use disorders with other forms of psychopathology may further intensify loneliness, as shared psychopathological factors could mediate or drive the link (Cacioppo & Hawkley, 2009; Montag, Wegmann, Sariyska, Demetrovics, & Brand, 2021). For instance, conditions like social anxiety or dysthymia might lead to both loneliness and problematic use of the internet, creating a reinforcing cycle between the two. Consequently, they may engage in online behaviors as a maladaptive form of emotional regulation. Therefore, it is possible that the type of internet-use disorder does not have a specific impact on the loneliness experienced by these individuals, and that loneliness is a complex, transdiagnostic construct. We did not identify a moderating effect of specific variables such as sex, age, and region, despite their established association with loneliness in previous meta-analyses (Wang & Zeng, 2024; Zhang et al., 2024). Their role should be further investigated in future high-quality studies.

Individuals with internet-use disorder symptoms seem to show greater loneliness than those without these conditions. Therefore, it may be useful to incorporate the assessment and management of loneliness in the treatment programs for internet-use disorders, training mental health professionals to address loneliness. Likewise, it may be advisable to guarantee the proactive engagement of these individuals in activities that promote social connection and social skills, such as support groups or community activities, volunteering or community services, and social group events, and maintaining active contact with friends and neighbors (Kim-Knauss, Degen, & Lang, 2024). Finally, it may be of interest to promote the inclusion of the family in the treatment plans when they can serve as a supportive network.

The main limitations of the evidence included in the present systematic review and meta-analysis were as follows: (1) Most studies (except three) had cross-sectional designs, making it not possible to explore causality in links between internet-use-disorder symptoms and loneliness; (2) All studies explored internet-use-disorder symptoms in community samples (not clinical samples) through self-report tools. Therefore, it is not possible to discuss these as mental disorders per se, but rather as features/symptoms of various internet-use disorders/concerns; (3) The use of self-report tools may be associated with biases, such as desirability bias, selection bias, and measurement error. Both loneliness and internet-use disorders/concerns are complex constructs, and using only one psychometric instrument to assess them does not capture their complex nature. In this vein, the UCLA-LS (the most widely used instrument to assess loneliness in the included studies) has faced criticism for not adequately capturing the complexity of the loneliness construct (Russell, 1996). Future studies could focus on developing tools to measure loneliness that address the limitations of current self-report instruments, such as the UCLA-LS; and (4) Indeed, alternative methodologies such as qualitative research or semi-structured interviews have been previously used to study the relationship between mental disorders and loneliness (Achterbergh et al., 2020; Birken et al., 2023) or the psychological experiences of loneliness in later life (Rees et al., 2023).

Some internet-use disorders/concerns, such as CSBD and PPU, were not represented in the results of this systematic review and meta-analysis, preventing a comprehensive understanding of these issues. While there are studies analyzing their associations with loneliness (Mestre-Bach & Potenza, 2023), none met the criteria to be included in the present systematic review and meta-analysis.

Future studies should explore internet-use disorders/concerns comprehensively using longitudinal designs and including clinical samples. Such approaches could help determine whether individuals who feel lonelier are more likely to develop these disorders/concerns, or if having an internet-use disorder/concern leads to negative consequences such as interpersonal conflicts, resulting in poor social support and increased loneliness. Moreover, our meta-regressions did not reveal significant effects of age, sex, sample size, or world region. This may suggest that the factors influencing the studied disorders/concerns are not strongly dependent on demographic variables like age or sex, potentially highlighting the role of individual psychological or personality features. The lack of an effect for sample size might indicate homogeneity in the results across studies or limitations in the available data. Future research should explore these variables further, considering larger and more diverse samples, as well as additional contextual and methodological factors, to better understand their potential influences.

## Conclusion

Individuals with versus without internet-use disorders/concerns exhibit more loneliness, although the effect size of this association is moderate. This relationship remains consistent across different internet-use disorders/concerns. These findings underscore the importance of addressing loneliness in the treatment of internet-use disorders/concerns.

## Supporting information

Mestre-Bach et al. supplementary material Mestre-Bach et al. supplementary material

## Supplementary material

The supplementary material for this article can be found at http://doi.org/10.1017/S0033291725000376.
