# Supplementary material for: The association between internet-use-disorder symptoms and loneliness: a systematic review and meta-analysis with a categorical approach
Source: Psychol Med. 2025 Mar 24;55:e77. doi: 10.1017/S0033291725000376 (PMC12080635; doi:10.1017/S0033291725000376)
Supplement: Mestre-Bach et al. supplementary material [file S0033291725000376sup001.docx]

**The Association Between Internet-Use Disorder Symptoms and Loneliness: A Systematic Review and Meta-Analysis with a Categorical Approach**

Supplemental material

# Index of supplementary tables

[*Table S1 Databases included in the biomedical sciences profile of UNIKA* 2](#_Toc189229168)

[*Table S2 Search query* 6](#_Toc189229169)

[*Table S3 Modified Newcastle-Ottawa Scale* 10](#_Toc189229170)

[*Table S4 Reports not retrieved* 11](#_Toc189229171)

[*Table S5 Origin of data of included studies* 13](#_Toc189229172)

[*Table S6 Socioeconomic and demographic characteristics and comorbidities from the overall group* 16](#_Toc189229173)

[*Table S7 Socioeconomic and demographic characteristics and comorbidities from the sample of individuals with internet use disorder symptoms* 21](#_Toc189229174)

[*Table S8 Socioeconomic and demographic characteristics and comorbidities from the samples of individuals without internet-use disorder symptoms* 25](#_Toc189229175)

[*Table S9 Description of samples of individuals with internet-use disorder symptoms* 28](#_Toc189229176)

[*Table S10 Description of samples of individuals at risk of internet-use disorders* 30](#_Toc189229177)

[*Table S11 Description of samples of individuals without internet-use disorders* 31](#_Toc189229178)

[*Table S12 Loneliness as a categorical variable* 33](#_Toc189229179)

[*Table S13 Final outcomes and effect sizes* 34](#_Toc189229180)

[*Table S14 Risk of bias evaluation (NOS)* 36](#_Toc189229181)

[*Table S15 Meta-regression analyses* 37](#_Toc189229182)

# Index of supplementary figures

[*Figure S1 Main analysis, funnel plot* 38](#_Toc189229183)

[*Figure S2 Main analysis, leave-one-out forest plot* 39](#_Toc189229184)

[*Figure S3 Analysis permitting a lower threshold for internet-use disorder symptoms* 40](#_Toc189229185)

[*Figure S4 Pooled average in the UCLA-LS for individuals without internet-use disorders* 41](#_Toc189229186)

[*Figure S5 Pooled average in the UCLA-LS for individuals with internet-use disorder symptoms* 42](#_Toc189229187)

[*Figure S6 Standardized mean difference between groups in the UCLA-LS* 43](#_Toc189229188)

# Supplementary tables

Table S1 Databases included in the biomedical sciences profile of UNIKA

| **Databases included in the biomedical sciences profile of the UNIKA Service from the University of Navarra (in alphabetical order)** |
| --- |
| 1. Academic Search Index (asx) |
| 2. AccessAnesthesiology |
| 3. AccessMedicine |
| 4. AccessPediatrics |
| 5. AccessScience |
| 6. AccessSurgery |
| 7. Ambrose Digital Library |
| 8. ASM Handbooks Online (edsaho) |
| 9. ASM Medical Materials Database |
| 10. ASM Micrograph Database |
| 11. BioOne Online Journals |
| 12. Books at JSTOR |
| 13. British Library Document Supply |
| Centre Inside Serials & Conference |
| Proceedings (edsbl) |
| 14. British Standards Online |
| 15. Business Source Complete |
| 16. Canadian Electronic Library |
| 17. Catálogo de la Biblioteca de la |
| Universidad de Navarra (cat00378a) |
| 18. Center for Research Libraries |
| 19. ChemSpider |
| 20. China/Asia On Demand |
| 21. CINAHL (cin20) |
| 22. CogPrints |
| 23. Credo Reference Collections (edscrc) |
| 24. DADUN (ir00048a) |
| 25. DASH |
| 26. Data-Planet Statistical Datasets & |
| Statistical Ready Reference |
| 27. Dialnet |
| 28. Directory of Open Access Journals |
| (edsdoj) |
| 29. eArticle |
| 30. eBook Academic Collection |
| (EBSCOhost) (e000xww) |
| 31. eBook Collection (EBSCOhost) |
| (nlebk) |
| 32. EconLit (ecn) |
| 33. EDS Foundation Index (eda) |
| 34. eLibro Premium |
| 35. ERIC (eric) |
| 36. eScholarship (edssch) |
| 37. EThOS |
| 38. EU Bookshop (edseub) |
| 39. European Union Open Data Portal |
| 40. Europeana |
| 41. Expanded Academic ASAP |
| 42. Films on Demand |
| 43. Fuente Académica Premier (fua) |
| 44. Gale Cengage Learning, Health & |
| Wellness Resource Center |
| 45. Gale Virtual Reference Library |
| 46. Gallica Bibliothèque Numérique |
| 47. Google Book Search (fe334f7c) |
| 48. GreenFILE (8gh) |
| 49. Harvard Library Bibliographic Dataset |
| (edshlc) |
| 50. HathiTrust (edshtl) |
| 51. Henry Stewart Talks |
| 52. HighWire Press (fa0f9666) |
| 53. Idunn.no |
| 54. IndianJournals.com |
| 55. Informit Health Collection (edsihc) |
| 56. Iprbooks |
| 57. JSTOR (fd43b2a1) |
| 58. JSTOR Life Sciences (edsjls) |
| 59. KERIS Theses & Dissertations |
| (edsker) |
| 60. Knigafund.ru (edskig) |
| 61. Korean Studies Information Service |
| System (KISS) (edskis) |
| 62. LexisNexis Academic: Law Reviews |
| (edslex) |
| 63. Maruzen eBook Library |
| 64. McGraw-Hill |
| 65. Medical Online |
| 66. Medical Online E-books |
| 67. Medical Online-E |
| 68. MEDLINE (cmedm) |
| 69. Minority Health Archive (edsuph) |
| 70. NARCIS |
| 71. Networked Digital Library of Theses & |
| Dissertations (edsndl) |
| 72. NORA (Norwegian Open Research |
| Archive) |
| 73. OAIster (edsoai) |
| 74. OJS vid Lunds Universitet (edsojs) |
| 75. Ovid Journals Full Text Medical |
| Research Database (fb0698e8) |
| 76. Oxford Bibliographies Online |
| 77. Oxford Clinical Psychology |
| 78. Oxford Handbooks Online (edsoho) |
| 79. Oxford Medicine Online |
| 80. Oxford Reference (edsoro) |
| 81. Oxford Scholarship Online (edsoso) |
| 82. ProQuest Dissertations and Theses (fb458d87) |
| 83. PsycARTICLES (edspdh) |
| 84. PsycBOOKS (edspzh) |
| 85. PsycCRITIQUES (edspvh) |
| 86. PsycheVisual |
| 87. Psychology and Behavioral Sciences |
| Collection (pbh) |
| 88. PsycINFO (psyh) |
| 89. Publisher Provided Full Text Searching |
| File (edb) |
| 90. PubMed Central (fd5a6824) |
| 91. R2 Digital Library |
| 92. RACO |
| 93. RECERCAT |
| 94. ReferenceSearch (edsref) |
| 95. RÖMPP Online |
| 96. SA ePublications Service |
| 97. SAGE Research Methods Datasets |
| 98. SAGE Video |
| 99. Scielo |
| 100. Scielo Books |
| 101. Science Citation Index (edswsc) |
| 102. ScienceDirect (edselp) |
| 103. Scopus |
| 104. Social Sciences Citation Index |
| (edswss) |
| 105. Springer Science+Business Media, |
| SpringerProtocols |
| 106. STAT!Ref |
| 107. Supplemental Index (edo) |
| 108. SveMed+ (edssmd) |
| 109. Torrossa |
| 110. TOXNET: GENETOX |
| 111. TOXNET: TOXLINE |
| 112. University Library Online -Университетская библиотека онлайн |
| 113. World Bank eLibrary (edswb |

Table S2 Search query

The search terms were determined as follows: (1) search for terms used in other published meta-analyses of related topics; (2) expansion of terms through the inclusion of synonyms and official terms used by the two major diagnostic manuals: DSM and ICD; (3) inclusion of MeSH terms and other terms that by themselves already indicated a behavioral addiction disorder (e.g. ludomania) (4) Terms indicating a pathological condition with those that indicated behavioral addictions were crossed with “AND” (i.e without the need of appearing one next to the other) in the case of terms related to addiction that were highly specific. (5)in the case of terms related to addiction that had multiple meanings (i.e., were less focused for our search), we combined each term on addiction with each term on conditions, tested them in PubMed and kept only those that appeared in its Phrase index(6) Additionally, some combinations were eliminated as they mostly signaled other types of problems or disorders (namely sexual violence).

**PubMed (Medline Plus) and UNIKA**

(lonely OR loneliness OR “social isolation” OR “socially isolated” OR singleness OR solitude) AND ( ( (Disorder OR disordered OR pathological OR pathologically OR compulsion OR compulsive OR compulsivity OR impulse OR impulsive OR impulsivity OR abuse OR abusive OR excess OR excessive OR abnormal OR problem OR problematic OR addictive OR addiction OR addict OR addicts) AND ((Gambling OR betting OR wagering OR punting OR Gambler OR gamblers) OR (Porn OR pornographic OR pornography OR erotic OR erotica OR x-rated OR “Adult films” OR “adult videos” OR “Adult film” OR “adult video” OR “adult content” OR “adult movie”) OR (buying OR shopping)) ) OR ( ludomania OR oniomania OR shopaholic OR overshopping OR hypersexual OR hypersexuality OR "behavior, addictive"[MeSH Terms] OR ”behavioural addiction” OR “behavioural addictions” OR “behavioral addiction” OR “behavioral addictions” OR “addictive behavior” OR “addictive behaviors” OR “addictive behaviour” OR “addictive behaviours” OR "internet abuse" OR “videogame abuse" OR “online compulsive" OR “sexual compulsivity" OR “game Disorder" OR “Gaming Disorder" OR “internet Disorder" OR “online impulse" OR “sexual impulse" OR “online impulsive" OR “sexual impulsivity" OR “online pathological" OR “Sex pathological" OR “sexual pathological" OR “internet abusive" OR “internet addict" OR “Sex addict" OR “sexual addict" OR “computer addiction" OR “game addiction" OR “Gaming addiction" OR “internet addiction" OR “online addiction" OR “Sex addiction" OR “sexual addiction" OR “videogame addiction" OR “computer addictive" OR “internet addictive" OR “online addictive" OR “game addicts" OR “Gaming addicts" OR “internet addicts" OR “Sex addicts" OR “sexual addicts" OR “sexual excess" OR “computer problem" OR “game problem" OR “internet problem" OR “online problem" OR “Sex problem" OR “sexual problem" OR “Gaming problematic" OR “internet problematic" OR “abuse Gaming" OR “compulsive computer" OR “compulsive Gaming" OR “compulsive internet" OR “compulsive online" OR “compulsive Sex" OR “compulsive sexual" OR “compulsivity sexual" OR “Disorder internet" OR “Disorder online" OR “Disorder Sex" OR “Disorder sexual" OR “disordered Gaming" OR “disordered internet" OR “disordered online" OR “disordered Sex" OR “disordered sexual" OR “impulsive Sex" OR “impulsive sexual" OR “pathological computer" OR “pathological game" OR “pathological Gaming" OR “pathological internet" OR “pathological online" OR “abnormal computer" OR “abnormal Sex" OR “abnormal sexual" OR “abusive internet" OR “addict computer" OR “addiction Gaming" OR “addiction internet" OR “addiction online" OR “addictive Gaming" OR “addictive internet" OR “addictive online" OR “addictive sexual" OR “excess Sex" OR “excessive computer" OR “excessive Gaming" OR “excessive internet" OR “excessive online" OR “excessive Sex" OR “excessive sexual" OR “problem computer" OR “problem game" OR “problem Gaming" OR “problem internet" OR “problem online" OR “problem sexual" OR “problem videogame" OR “problematic computer" OR “problematic game" OR “problematic Gaming" OR “problematic internet" OR “problematic online" OR “problematic sexual" OR “problematic videogame") )

**Web of Science:**

(AB= (lonely OR loneliness OR “social isolation” OR “socially isolated” OR singleness OR solitude) AND ( ( (Disorder OR disordered OR pathological OR pathologically OR compulsion OR compulsive OR compulsivity OR impulse OR impulsive OR impulsivity OR abuse OR abusive OR excess OR excessive OR abnormal OR problem OR problematic OR addictive OR addiction OR addict OR addicts) AND ((Gambling OR betting OR wagering OR punting OR Gambler OR gamblers) OR (Porn OR pornographic OR pornography OR erotic OR erotica OR x-rated OR “Adult films” OR “adult videos” OR “Adult film” OR “adult video” OR “adult content” OR “adult movie”) OR (buying OR shopping)) ) OR ( ludomania OR oniomania OR shopaholic OR overshopping OR hypersexual OR hypersexuality OR "behavior, addictive" OR ”behavioural addiction” OR “behavioural addictions” OR “behavioral addiction” OR “behavioral addictions” OR “addictive behavior” OR “addictive behaviors” OR “addictive behaviour” OR “addictive behaviours” OR "internet abuse" OR “videogame abuse" OR “online compulsive" OR “sexual compulsivity" OR “game Disorder" OR “Gaming Disorder" OR “internet Disorder" OR “online impulse" OR “sexual impulse" OR “online impulsive" OR “sexual impulsivity" OR “online pathological" OR “Sex pathological" OR “sexual pathological" OR “internet abusive" OR “internet addict" OR “Sex addict" OR “sexual addict" OR “computer addiction" OR “game addiction" OR “Gaming addiction" OR “internet addiction" OR “online addiction" OR “Sex addiction" OR “sexual addiction" OR “videogame addiction" OR “computer addictive" OR “internet addictive" OR “online addictive" OR “game addicts" OR “Gaming addicts" OR “internet addicts" OR “Sex addicts" OR “sexual addicts" OR “sexual excess" OR “computer problem" OR “game problem" OR “internet problem" OR “online problem" OR “Sex problem" OR “sexual problem" OR “Gaming problematic" OR “internet problematic" OR “abuse Gaming" OR “compulsive computer" OR “compulsive Gaming" OR “compulsive internet" OR “compulsive online" OR “compulsive Sex" OR “compulsive sexual" OR “compulsivity sexual" OR “Disorder internet" OR “Disorder online" OR “Disorder Sex" OR “Disorder sexual" OR “disordered Gaming" OR “disordered internet" OR “disordered online" OR “disordered Sex" OR “disordered sexual" OR “impulsive Sex" OR “impulsive sexual" OR “pathological computer" OR “pathological game" OR “pathological Gaming" OR “pathological internet" OR “pathological online" OR “abnormal computer" OR “abnormal Sex" OR “abnormal sexual" OR “abusive internet" OR “addict computer" OR “addiction Gaming" OR “addiction internet" OR “addiction online" OR “addictive Gaming" OR “addictive internet" OR “addictive online" OR “addictive sexual" OR “excess Sex" OR “excessive computer" OR “excessive Gaming" OR “excessive internet" OR “excessive online" OR “excessive Sex" OR “excessive sexual" OR “problem computer" OR “problem game" OR “problem Gaming" OR “problem internet" OR “problem online" OR “problem sexual" OR “problem videogame" OR “problematic computer" OR “problematic game" OR “problematic Gaming" OR “problematic internet" OR “problematic online" OR “problematic sexual" OR “problematic videogame") )

OR TI=(AB= (lonely OR loneliness OR “social isolation” OR “socially isolated” OR singleness OR solitude) AND ( ( (Disorder OR disordered OR pathological OR pathologically OR compulsion OR compulsive OR compulsivity OR impulse OR impulsive OR impulsivity OR abuse OR abusive OR excess OR excessive OR abnormal OR problem OR problematic OR addictive OR addiction OR addict OR addicts) AND ((Gambling OR betting OR wagering OR punting OR Gambler OR gamblers) OR (Porn OR pornographic OR pornography OR erotic OR erotica OR x-rated OR “Adult films” OR “adult videos” OR “Adult film” OR “adult video” OR “adult content” OR “adult movie”) OR (buying OR shopping)) ) OR ( ludomania OR oniomania OR shopaholic OR overshopping OR hypersexual OR hypersexuality OR "behavior, addictive" OR ”behavioural addiction” OR “behavioural addictions” OR “behavioral addiction” OR “behavioral addictions” OR “addictive behavior” OR “addictive behaviors” OR “addictive behaviour” OR “addictive behaviours” OR "internet abuse" OR “videogame abuse" OR “online compulsive" OR “sexual compulsivity" OR “game Disorder" OR “Gaming Disorder" OR “internet Disorder" OR “online impulse" OR “sexual impulse" OR “online impulsive" OR “sexual impulsivity" OR “online pathological" OR “Sex pathological" OR “sexual pathological" OR “internet abusive" OR “internet addict" OR “Sex addict" OR “sexual addict" OR “computer addiction" OR “game addiction" OR “Gaming addiction" OR “internet addiction" OR “online addiction" OR “Sex addiction" OR “sexual addiction" OR “videogame addiction" OR “computer addictive" OR “internet addictive" OR “online addictive" OR “game addicts" OR “Gaming addicts" OR “internet addicts" OR “Sex addicts" OR “sexual addicts" OR “sexual excess" OR “computer problem" OR “game problem" OR “internet problem" OR “online problem" OR “Sex problem" OR “sexual problem" OR “Gaming problematic" OR “internet problematic" OR “abuse Gaming" OR “compulsive computer" OR “compulsive Gaming" OR “compulsive internet" OR “compulsive online" OR “compulsive Sex" OR “compulsive sexual" OR “compulsivity sexual" OR “Disorder internet" OR “Disorder online" OR “Disorder Sex" OR “Disorder sexual" OR “disordered Gaming" OR “disordered internet" OR “disordered online" OR “disordered Sex" OR “disordered sexual" OR “impulsive Sex" OR “impulsive sexual" OR “pathological computer" OR “pathological game" OR “pathological Gaming" OR “pathological internet" OR “pathological online" OR “abnormal computer" OR “abnormal Sex" OR “abnormal sexual" OR “abusive internet" OR “addict computer" OR “addiction Gaming" OR “addiction internet" OR “addiction online" OR “addictive Gaming" OR “addictive internet" OR “addictive online" OR “addictive sexual" OR “excess Sex" OR “excessive computer" OR “excessive Gaming" OR “excessive internet" OR “excessive online" OR “excessive Sex" OR “excessive sexual" OR “problem computer" OR “problem game" OR “problem Gaming" OR “problem internet" OR “problem online" OR “problem sexual" OR “problem videogame" OR “problematic computer" OR “problematic game" OR “problematic Gaming" OR “problematic internet" OR “problematic online" OR “problematic sexual" OR “problematic videogame") )

Table S3 Modified Newcastle-Ottawa Scale

An asterisk indicates that the option gives a star (low risk of bias)

| **Selection** |
| --- |
| Representativeness of the sample: a) Truly representative of the average in the target population. * (all subjects or random sampling) b) Somewhat representative of the average in the target population. * (non-random sampling) c) Selected group of users d) No description of the sampling strategy. |
| 2) Sample size: a) Justified and satisfactory. *  b) Not justified. |
| 3) Non-respondents: a) Comparability between respondents and non-respondents characteristics is established, and the response rate is satisfactory. * b) The response rate is unsatisfactory, or the comparability between respondents and non-respondents is unsatisfactory. c) No description of the response rate or the characteristics of the responders and the non-responders. |
| 4) Ascertainment of INTERNET USE DISORDER: a) Validated measurement tool: CLINICAL DIAGNOSIS. * b) RECORD LINKAGE*  c) SELF REPORT OR QUESTIONNAIRE |
| **Comparability** |
| 1) The subjects in different outcome groups are comparable, based on the study design or analysis. Confounding factors are controlled. a) The study controls for AGE. * OR b) The study controls for SEX. * |
| **Outcome**  1) Statistical test: a) The statistical test used to analyze the data is clearly described and appropriate, and the measurement of the association is presented, including confidence intervals and the probability level (p value) OR ALL NUMBERS OF THE 2X2 MATRIX ARE PRESENTED (CALCULATION BY PERCENTAGES DOES NOT COUNT) * b) The statistical test is not appropriate, not described or incomplete. |

Table S4 Reports not retrieved

Studies for which we were not able to locate the full text after 1-Searching our institutional databases, 2-Searching online for access (including the journal website), 3-asking for an institutional inter-library loan.

| ***Reference*** |
| --- |
| Dai, J., Zhao, Z., Dong, H., Du, X., & Guang-Heng, D. (2024). The severity of addiction mediates loneliness and cortical volume in internet gaming disorder. *NeuroReport*, *35*(1), 61–70. https://doi.org/10.1097/WNR.0000000000001975 |
| Fioravanti, G., Dèttore, D., & Casale, S. (2012). Adolescent Internet Addiction: Testing the Association Between Self-Esteem, the Perception of Internet Attributes, and Preference for Online Social Interactions. *Cyberpsychology, Behavior, and Social Networking*, *15*(6), 318–323. https://doi.org/10.1089/cyber.2011.0358 |
| Ghassemzadeh, L., Shahraray, M., & Moradi, A. (2008). Prevalence of Internet Addiction and Comparison of Internet Addicts and Non-Addicts in Iranian High Schools. *CyberPsychology & Behavior*, *11*(6), 731–733. https://doi.org/10.1089/cpb.2007.0243 |
| Goh, Z. H., Tandoc, E. C., & Chan, V. X. (2023). Alone and lonely? How physical and perceived isolation can lead to problematic internet use. *Behaviour & Information Technology*, *42*(15), 2588–2600. https://doi.org/10.1080/0144929X.2022.2134825 |
| Huan, V. S., Ang, R. P., Chong, W. H., & Chye, S. (2014). The Impact of Shyness on Problematic Internet Use: The Role of Loneliness. *The Journal of Psychology*, *148*(6), 699–715. https://doi.org/10.1080/00223980.2013.825229 |
| Isiklar, A. (2012). Examination of the variables predicting internet addiction in adolescence |
| Kim, B.-N., & Ko, H. (2020). Psychometric Properties of the Nine-Item Korean Internet Gaming Disorder Scale: Short Form. *Cyberpsychology, Behavior, and Social Networking*, *23*(12), 854–859. https://doi.org/10.1089/cyber.2020.0227 |
| Kitiş, Y., Dağci, B., Köse, N., & Geniş, Ç. (2022). The use of social media among high school students and its relationship with the perception of loneliness: A pilot study. *Journal of Child and Adolescent Psychiatric Nursing*, *35*(4), 341–348. https://doi.org/10.1111/jcap.12388 |
| Koc, M., & Gulyagci, S. (2013). Facebook Addiction Among Turkish College Students: The Role of Psychological Health, Demographic, and Usage Characteristics. *Cyberpsychology, Behavior, and Social Networking*, *16*(4), 279–284. https://doi.org/10.1089/cyber.2012.0249 |
| Koyuncu, T., Unsal, A., Arslantas, D. Assessment of internet addiction and loneliness in secondary and high school students. *J Pak Med Assoc*, 64(9)998-1002 |
| Kuem, J., Ray, S., Hsu, P.-F., & Khansa, L. (2021). Smartphone Addiction and Conflict: An Incentive-Sensitisation Perspective of Addiction for Information Systems. *European Journal of Information Systems*, *30*(4), 403–424. https://doi.org/10.1080/0960085X.2020.1803154 |
| Lee, K.-T., Noh, M.-J., & Koo, D.-M. (2013). Lonely People Are No Longer Lonely on Social Networking Sites: The Mediating Role of Self-Disclosure and Social Support. *Cyberpsychology, Behavior, and Social Networking*, *16*(6), 413–418. https://doi.org/10.1089/cyber.2012.0553 |
| Tefertiller, A. C., & Maxwell, L. C. (2023). Is it all binge-watching? Viewing patterns, audience activity, psychological antecedents, and media addiction in extended-time television viewing. *The Social Science Journal*, 1–15. https://doi.org/10.1080/03623319.2022.2151795  Tefertiller, A. C., & Maxwell, L. C. (2023). Is it all binge-watching? Viewing patterns, audience activity, psychological antecedents, and media addiction in extended-time television viewing. *The Social Science Journal*, 1–15. https://doi.org/10.1080/03623319.2022.2151795 |
| Tian, Y., Guo, Z. X., Shi, J. R., Bian, Y. L., Han, P. G., Wang, P., & Gao, F. Q. (2018). Bidirectional Mediating Role of Loneliness in the Association Between Shyness and Generalized Pathological Internet Use in Chinese University Students: A Longitudinal Cross-Lagged Analysis. *The Journal of Psychology*, *152*(8), 529–547. https://doi.org/10.1080/00223980.2018.1468309 |
| Whang, L. S.-M., Lee, S., & Chang, G. (2003). Internet Over-Users’ Psychological Profiles: A Behavior Sampling Analysis on Internet Addiction. *CyberPsychology & Behavior*, *6*(2), 143–150. https://doi.org/10.1089/109493103321640338 |
| Zhang, B., Liang, H., Luo, Y., Peng, Y., Qiu, Z., Mao, H., Yuan, M., & Xiong, S. (2022). Loneliness and mobile phone addiction in Chinese college students: a moderated mediation model. *Journal of Psychology in Africa*, *32*(6), 605–610. https://doi.org/10.1080/14330237.2022.2121474 |

Table S5 Origin of data of included studies

In the case of studies published after 2020, if no data collection date was provided and no mention of COVID-19 was included in the manuscript, we assumed that they had been carried out previous to the onset of the COVID-19. Otherwise, we assumed that they had been carried out after the onset of the pandemic.

| **ID** | **After COVID (2020)** | **Date of data collection** | **Origin of data** |
| --- | --- | --- | --- |
| Akbari 2023 | Yes | Between September 22, 2021 and October 20, 2021 | Adolescents from Tehran, Iran.  145 were seventh grade, 202 were eighth grade, 645 were first secondary grade, 752 were second secondary grade, 252 were third secondary grade, 233 were fourth secondary grade, and 161 were first year undergraduates. |
| Aktepe 2013 | No |  | High schools in the city center of Isparta |
| Erol 2019 | No |  | Participants were college students registered to a public university located in the Mediterranean region of Turkey |
| Hardie 2007 | No |  | Online survey. Participants were recruited through internet chat rooms, online forums and networks known to the researchers. |
| Hou 2019 | No |  | Participants were recruited by putting up a poster at campus and advertising on the Internet at Anhui University in China. |
| Jeon 2022 | No |  | Data observed for 3 years among longitudinal panel data for the Korean Adolescent Game User Cohort Research were analyzed to track the adolescents before they graduated from secondary school. The panel survey was conducted by the Korea Creative Content Agency (KOCCA) |
| Karaibrahimoglu 2023 | Unclear | December 2021 | Freshman and senior students of the Faculty of Economics and Administrative Sciences of a public university. |
| King 2013 | No | From June to August 2012. | Fifty secondary schools in the outer metropolitan region of Adelaide, South Australia, were randomly selected from a comprehensive list of public and private schools. In total, seven coeducational schools (four public, three private) provided consent to participate. |
| Lin 2024 | No |  | Data was derived from the Taiwan Youth Project. The first phase began in 2000 and included annual interviews over nine years. In the second phase, participants who had responded to the last survey in the first phase were recruited again (response rate: 74 %) and then surveyed in 2011, 2014, and 2017. Participants in the last wave (2017) were in their early 30s (mean age=30.3). |
| Liu 2023 | Yes | Between April and October of 2022 | Nursing postgraduate students from five universities in different regions of China. |
| Myrseth 2017 | No |  | Study carried out on Norwegian Armed Forces: approximately 50% of the conscripts were randomly selected from the pool of conscripts who started their first year of military service in Norway. |
| Orsolini 2023 | Yes | Time frames from 25 January 2021 to 26 February 2021 and from 2 December 2021 to 23 February 2022. | Italian young people (aged 18–35), using a snowball sampling strategy. |
| Paschke 2022 | Yes | Between May 19 and June 06, 2021 | Online survey by German market research and opinion polling company Forsa. 1,128 households with children between 10 and 17 years and a respective parent were included. The sample was representative regarding residence region, age, and gender |
| Reed 2015 | No |  | Participants were recruited through links posted on internet sites (social networking sites, blogging and microblogging sites, and gaming sites) |
| Sangram 2020 | No |  | Undergraduate students of arts, science, and commerce streams falling in the age range of 17–20 years, of St. Xavier’s College of Arts, Science and Commerce, Goa. |
| Shettar 2017 | No |  | Post-graduate students from Yenepoya University through simple random sampling, Mangaluru, located in Southern India. |
| Shi 2017 | No |  | Participants from eight middle or high schools (110 classes) in Beijing, China. |
| Smith 2022 | Yes | During the academic year 2020–2021 | Participants were students from the Caribbean based University of Trinidad and Tobago (UTT). |
| Van Rooij 2014 | No | The study aggregates the 2009, 2010 and 2011 samples. | Samples of the yearly Dutch Monitor Study ‘Internet and Youth’. In 2009, ten schools participated (4909 questionnaires were distributed), ten schools participated in 2010 (4133 distributed) and 13 schools participated in 2011 (3756 distributed) |
| Verma 2023 | Unclear | February 2020. | Undergraduate students in a Medical College of North India between 18 and 25 years of age were recruited through a Google forms that was shared on the WhatsApp group of all five batches of students (MBBS I, II, III, IV, and interns). |
| Wang 2022 | Yes |  | A self-administered, structured questionnaire in Chinese was distributed to participants from three universities of Guangdong (China) through an online questionnaire platform during regular school hours. The data used in this study was obtained from an ongoing longitudinal study in Guangdong, China. |
| Yu 2022 | No | From April to July 2018 | In Shanghai in East China and Xi’an in West China. All the first-year students (the seventh year of formal education) of eight conveniently selected secondary schools (five in Shanghai and three in Xi’an). |
| Zakaria 2023 | No | Between February to May 2016. | Students from the faculties of dentistry, medicine, optometry, pharmacy, and psychology of University Kebangsaan Malaysia (UKM), a public university in Malaysia. |

Table S6 Socioeconomic and demographic characteristics and comorbidities from the overall group

| **ID** | **Socioeconomic and demographic characteristics** | **Ethnicity** | **Comorbidities** |
| --- | --- | --- | --- |
| Akbari 2023 | Education level: 145 were seventh grade, 202 were eighth grade, 645 were first secondary grade, 752 were second secondary grade, 252 were third secondary grade, 233 were fourth secondary grade, and 161 were first year undergraduates. |  |  |
| Aktepe 2013 | SES was controlled by cluster sampling of schools from different socioeconomic levels. The students were given a survey form concerning sociodemographic factors: age, gender, family structure; their parents’ education levels. |  | 36.6% of the adolescents (n=602) were identified as committing Self-injurious behavior (SIB) within the last six months, as follows: 34.1% (n=561) committed SIB 1-5 times, while 2.5% of them (n=41) did so 6 or more times. Insomnia was present in 30.8% of cases. |
| Erol 2019 | Data were collected across all class levels. Data were collected across all class levels, with 36.5% (n = 177) as freshmen, 23.9% (n = 116) as sophomores, 22.5% (n = 109) as juniors, and 17.1% (n = 84) as senior students. |  |  |
| Hardie 2007 | Education level: 25% had completed secondary school level, 5% had trade or vocational qualifications, 30% had incomplete tertiary qualifications, 25% had completed a tertiary degree and 15% had postgraduate qualifications.  Employment status: 10% were unemployed, 19% had part-time employment, 31% were students, and 40% were in full-time employment. |  |  |
| Hou 2019 |  |  | Healthy, with normal or corrected-to-normal vision, no history of neurological or psychiatric disorder. |
| Jeon 2022 | Education level from all Participants (n = 778): Elementary school: 287 (36.9%) Middle school: 273 (35.1%) High school: 218 (28.0%) |  | All Participants (n = 778) Anxiety: Mean = 0.722, SD = 0.479 Pathological Gaming: Mean = 3.148, SD = 1.042 |
| Karaibrahimoglu 2023 | Monthly income:  0-499 ₺ 64 (%14.3) 500-1499 ₺ 138 (%30.7) 1500-3000 ₺ 130 (%28.9) >3000 ₺ 117 (%26.1) Family tipe nuclear 352 (%78.4); Wide 73 (%16.3); Divorced 24 (%5.3) Who he/she lives with: Family 171 (%38.1); Friend 132 (%29.4); Alone 146 (%32.5) |  |  |
| King 2013 | A standardised questionnaire assessed basic demographic information (i.e. age, sex, school, grade, ethnic background, main language spoken at home) |  |  |
| Lin 2024 | Parent’s education: highest education level among parents in years 11.57 (SD=3.01) |  | Substance use 8.22% Depressive symptoms (range:1–5) 0.49 (0.49) |
| Liu 2023 | Only chilld family: 27.60% |  |  |
| Myrseth 2017 | Education level: 96% had completed high school whereas only 2.3% had higher education (after high school). |  | Significant correlations con Gaming: Weekly time spent gaming .435; Loneliness .210; BP-Lack of external stimulation .190; Depression .257; Anxiety .199 |
| Orsolini 2023 | The mean years of educational level was 16.2 (SD = 1.7). |  | Overall, the sample displayed a severe general psychopathology in 49.4% of the participants, particularly, most participants showed a severe-to-extremely-severe depressive symptomatology (49.4%; n = 813), a severe-to-extremely-severe anxiety symptomatology (49.0%; n = 804), and severe-to-extremely-severe stress symptomatology (48.6%; n = 800).  RESULTS of DASS-21: Depression: Normal: 33.4%; Mild: 10.9%; Moderate: 17.1%; Severe: 18.9%; Extremely Severe: 19.8%. Anxiety: Normal: 51.1%; Mild: 11.4%; Moderate: 15.1%; Severe: 13.6%; Extremely Severe: 8.8%. Stress: Normal: 21.8%; Mild: 10.5%; Moderate: 19.1%; Severe: 27.4%; Extremely Severe: 21.2%. |
| Paschke 2022 | Education Level: High: 504 [56.12% (95% CI: 52.86–59.34)], Medium: 325 [36.19% (95% CI: 33.11–39.39)], Low: 69 [7.68% (95% CI: 6.12–9.61)]. Occupation: Full-time employment/school attendance: 910 [95.49% (95% CI: 93.98–96.63)], Part-time employment/apprenticeship: 32 [3.36% (95% CI: 2.39–4.70)], Other: 11 [1.15% (95% CI: 0.65–2.05)]. |  | GAD-2 sum score 0.92 (1.34; 0–6) (anxiety) ISI sum score 8.54 (6.45; 0–28) (insomnia) PHQ-9 sum score 4.69 (4.94; 0–27) (depression) |
| Reed 2015 | Relationship status: 305 (60%) single, 65 (13%) married or in a civil partnership; 105 (21%) in other forms of relationship; and 30 (6%) divorced or widowed | 202 (40%) White; 50 (10%) Mixed/Multiple Ethnic Groups; 141 (28%) Asian; 106 (21%) Black/African/Caribbean; and 6 (1%) Other Ethnic Group. | 25% depressión, 24,4% anxiety |
| Sangram 2020 | Of 200 students 55 (27.5%) belonged to arts, 78 (39%) belonged to science and 67 (33.5%) belonged to commerce faculty.  Class-wise classification of 1st, 2nd, and 3rd year students were as follows 84 (42%), 63 (31.5%), and 53 (26.5%). |  |  |
| Shettar 2017 | Relationship status: 46 were married (46%) and 54 were single (54%); 82% were from nuclear family and 17% were from joint family and 1% were from extended family Degree type: 69% of the participants belonged to medical post-graduation and 31% belonged to dental postgraduation. |  |  |
| Shi 2017 | Grade 7 (N= 605, Mage = 13.588, SD = 0.465), grade 8 (N = 607, Mage = 14.529, SD = 0.484), grade 10 (N = 1028, Mage = 16.554, SD = 0.433), and grade 11 (N = 759, Mage = 17.444, SD = 0.395) |  |  |
| Smith 2022 |  |  |  |
| Van Rooij 2014 |  |  | Non-Online Players vs. Online Players (Note: Online players are not identified as addicted or having problematic use)  Online players:  - Alcohol use: 36.43%  - Smoking: 34.62%  - Depressive mood: 2.16 (0.70)  - Social anxiety (general): 1.71 (0.71)  - Social anxiety (new situations): 2.27 (0.78)  - Negative self-esteem: 1.68 (0.52)   Non-online players:  - Alcohol use: 63.57%  - Smoking: 65.38%  - Depressive mood: 2.16 (0.70)  - Social anxiety (general): 1.71 (0.71)  - Social anxiety (new situations): 2.27 (0.78)  - Negative self-esteem: 1.68 (0.52) |
| Verma 2023 |  |  |  |
| Wang 2022 | Regarding parents’ education level, 60.5% of fathers and 71.7% of mothers have less than or equal to 9 years of education. As for residence, more than half of the students (54.6%) were from rural areas |  |  |
| Yu 2022 | 54.1% self-reported good or very good family socio-economic status. About half of the participants’ fathers (53.9%) and mothers (53.6%) had attended college or a higher education level.  Poor SES: 38.9%, Good SES: 54.1, Missing information: 7.0% |  |  |
| Zakaria 2023 |  | Malays (59.4%), Chinese (25.8%), Indian (11.3%), and other ethnic groups (3.5%) | Alcohol: total 12.9%, boys 19,3%, girls 7,1% Substanse use: total 2,7%, boys 3,3%, girls 2,1% |

Table S7 Socioeconomic and demographic characteristics and comorbidities from the sample of *individuals with internet use disorder symptoms*

| **ID** | **Socioeconomic and demographic characteristics** | **Ethnicity** | **Comorbidities** |
| --- | --- | --- | --- |
| Akbari 2023 |  |  | PG (Problematic gambling): Social anxiety symptoms 31.05 (0.85)/ ADHD symptoms 8.99 (0.16) Problematic social media use with gaming disorder Social anxiety symptoms 41.10 (0.66)/ ADHD symptoms 10.12 (0.12) Disordered gambling with problematic social media. Social anxiety symptoms 40.62 (1.51)/ ADHD symptoms 10.15 (0.27) |
| Aktepe 2013 | No correlation was found between the prevalence of PIA and schools of low (n=71, 14.7%), middle (n=83, 14.2%), or high (n=83, 14.4%) socioeconomic levels (χ2 = 0.055, p=0.973). |  | Self-injurious behavior within the last six months 53.6%. Insomnia: 40% |
| Erol 2019 |  |  |  |
| Hardie 2007 |  |  | Excessive users: (n = 50) Social Anxiety 19.76 (4.48)  Users with internet addiction: (n = 8) Social Anxiety** 22.25 (5.52) |
| Hou 2019 |  |  |  |
| Jeon 2022 | Education level from Risk Group (n = 355): Elementary school: 127 (35.8%) Middle school: 120 (33.8%) High school: 108 (30.5%) |  | Risk Group (n = 355) Anxiety: Mean = 0.900, SD = 0.352 Pathological Gaming: Mean = 3.567, SD = 0.557 |
| Karaibrahimoglu 2023 |  |  |  |
| King 2013 |  |  | 3 groups:  1. VG Only (Pathological Video Gaming): - Depression: 60.9 ± 19.6  - OCD: 51.3 ± 16.7  - Panic: 60.8 ± 21.3  - Separation Anxiety: 59.6 ± 21.8  - Social Anxiety: 44.9 ± 14.1  - Anxiety Total: 52.0 ± 19.0   2. PIU Only (Pathological Internet Use):  - Depression: 67.1 ± 22.6  - OCD: 57.6 ± 16.6  - Panic: 66.1 ± 23.7  - Separation Anxiety: 67.1 ± 24.9  - Social Anxiety: 54.9 ± 13.2  - Anxiety Total: 61.0 ± 19.6   3. PVG and PIU (Co-occurring):  - Depression: 66.2 ± 20.1  - OCD: 55.7 ± 15.2  - Panic: 70.9 ± 23.3  - Separation Anxiety: 71.2 ± 24.8  - Social Anxiety: 51.8 ± 14.1  - Anxiety Total: 60.7 ± 19.7 |
| Lin 2024 | Parent’s education: highest education level among parents in years 11.41 (2.79) |  | Substance use 9.01% Depressive symptoms (range:1–5) 0.62 (0.58) |
| Liu 2023 | Education type: median 85 master |  |  |
| Myrseth 2017 |  |  | Gaming addiction was most strongly correlated with weekly time spent gaming (.435), but was also significantly correlated with depression (.257), loneliness (.210), anxiety (.199). |
| Orsolini 2023 |  |  | RESULTS of DASS-21: Depression: Normal: 13.9%; Mild: 8.9%; Moderate: 19.4%; Severe: 19.9%; Extremely Severe: 38.0%. Anxiety: Normal: 34.3%; Mild: 16.2%; Moderate: 17.0%; Severe: 17.8%; Extremely Severe: 14.7%. Stress: Normal: 8.9%; Mild: 8.1%; Moderate: 17.5%; Severe: 30.6%; Extremely Severe: 23.3%. |
| Paschke 2022 |  |  | GAD-2 sum score 2.79 (0.31)/ PHQ-9 sum score 2.44 (0.99) / ISI sum score 13.05 (1.02) |
| Reed 2015 |  |  | 38,5% depressión, 39,6% anxiety |
| Sangram 2020 |  |  |  |
| Shettar 2017 |  |  |  |
| Shi 2017 |  |  |  |
| Smith 2022 |  |  |  |
| Van Rooij 2014 |  |  | In the sample of Online Players, participants completed the VAT (Video Game Addiction Test). Table 2 of the article categorizes the data by gender (boys/girls) and by low or high VAT scores.   Boys with low VAT scores:  - Alcohol: 92.63%  - Smoking: 92.33%  - Cannabis: 89.19%  - Depressive mood: 2.03 (0.64)  - Social anxiety (general): 1.65 (0.65)  - Social anxiety (new situations): 2.16 (0.73)   Boys with high VAT scores:  - Alcohol: 7.37%  - Smoking: 7.67%  - Cannabis: 10.81%  - Depressive mood: 2.03 (0.64)  - Social anxiety (general): 1.65 (0.65)  - Social anxiety (new situations): 2.16 (0.73)   Girls with low VAT scores:  - Alcohol: 97.11%  - Smoking: 98.30%  - Cannabis: 90.32%  - Depressive mood: 2.31 (0.72)  - Social anxiety (general): 1.58 (0.51)  - Social anxiety (new situations): 2.43 (0.81)   Girls with high VAT scores:  - Alcohol: 2.89%  - Smoking: 1.70%  - Cannabis: 9.68%  - Depressive mood: 3.24 (0.79)  - Social anxiety (general): 2.68 (1.14)  - Social anxiety (new situations): 2.89 (0.95) |
| Verma 2023 |  |  |  |
| Wang 2022 |  |  | Social Anxiety Disorder and depressive symptoms were statistically significant risk factors for PSU. |
| Yu 2022 |  |  |  |
| Zakaria 2023 |  | Malay 99 (61.9%) | ADHD (Inattention) 71,2% / ADHD (Hyperactivity) 54,4% Depression 55% Anxiety 71,9% Stress 53,1% |

Table S8 Socioeconomic and demographic characteristics and comorbidities from the samples of individuals without internet-use disorder symptoms

| **ID** | **Socioeconomic and demographic characteristics** | **Ethnicity (list type and %)** | **Comorbidities** |
| --- | --- | --- | --- |
| Akbari 2023 |  |  | Social anxiety symptoms 31.53 (0.28)/ ADHD symptoms 8.45 (0.05) |
| Aktepe 2013 |  |  | Self-injurious behavior within the last six months 33.7%. Insomnia 29.3% |
| Erol 2019 |  |  |  |
| Hardie 2007 |  |  | Average users: Social Anxiety** 16.95 (5.15) |
| Hou 2019 |  |  |  |
| Jeon 2022 | Education level from Non-Risk Group (n = 327): Elementary school: 118 (36.1%) Middle school: 121 (37.0%) High school: 88 (26.9%)​ |  | Non-Risk Group (n = 327) Anxiety: Mean = 0.250, SD = 0.293 Pathological Gaming: Mean = 1.528, SD = 0.687​ |
| Karaibrahimoglu 2023 |  |  |  |
| King 2013 |  |  | Depression: 54.7 ± 17.4  OCD: 48.9 ± 12.6  Panic: 55.8 ± 17.5  Separation Anxiety: 57.6 ± 18.8  Social Anxiety: 47.2 ± 12.6  Anxiety Total: 50.2 ± 15.8 |
| Lin 2024 | Parent's education: highest education level among parents in years 11.60 (3.04) |  | Substance use 8,11% Depressive symptoms (range:1–5) 0.47 (0.47) |
| Liu 2023 | Education type: median 55 master, 49.5 phd |  |  |
| Myrseth 2017 |  |  |  |
| Orsolini 2023 |  |  | RESULTS of DASS-21: Depression: Normal: 39.3%; Mild: 11.5%; Moderate: 16.4%; Severe: 18.6%; Extremely Severe: 14.3%. Anxiety: Normal: 56.1%; Mild: 9.9%; Moderate: 14.5%; Severe: 12.4%; Extremely Severe: 7.1%. Stress: Normal: 25.7%; Mild: 11.2%; Moderate: 19.5%; Severe: 26.5%; Extremely Severe: 17.1%. |
| Paschke 2022 |  |  | PHQ-9 sum score 4.3 (0.15)/ GAD-2 sum score 0.83 (0.04)/ ISI sum score 8.32 (0.21) |
| Reed 2015 |  |  |  |
| Sangram 2020 |  |  |  |
| Shettar 2017 |  |  |  |
| Shi 2017 |  |  |  |
| Smith 2022 |  |  |  |
| Van Rooij 2014 |  |  | Alcohol use 63.57% Smoking 65.38% 44.62% Depressive mood 2.16 (0.70) Social anxiety (generaL) 1.71 (0.71) Social anxiety (new situations) 2.27 (0.78) |
| Verma 2023 |  |  |  |
| Wang 2022 |  |  |  |
| Yu 2022 |  |  |  |
| Zakaria 2023 |  | Malay 186(65.3%) | ADHD (Inattention) 48,5% / ADHD (Hyperactivity) 33,1% Depression 32,7% Anxiety 51,9% Stress 31% |

Table S9 Description of samples of individuals with internet-use disorder symptoms

N is the sample size of the group or groups with internet-use disorder symptoms within a study, sd is standard deviation. DG: disordered gaming, PG: problematic gambling, PUSM: Problematic use of social media, GD: gambling disorder, PUI: problematic use of the internet, PVG: problematic video gaming. * indicates that the two lines share the same subjects. When multiple samples from the same study are described, we have included additional details (following author´s descriptions) to differentiate them.

|  | **n** | **Mean age in years** | **Sd age** | **Gender (% female)** | **Loneliness** | **Sd loneliness** |
| --- | --- | --- | --- | --- | --- | --- |
| Akbari 2023: DG&PUSM | 34 |  |  |  | 7.32 | 1.75 |
| Akbari 2023: PG | 183 |  |  |  | 6.09 | 2.03 |
| Akbari 2023: PUSM&GD | 407 |  |  |  | 7.3 | 2.22 |
| Aktepe 2013 | 237 |  |  | 61.18 | 20.25 | 7.65 |
| Erol 2019 | 16 |  |  |  | 38.8 | 8.78 |
| Hardie 2007: emotional loneliness | 8 |  |  |  | 13 | 5.68 |
| Hardie 2007: social loneliness | 8 |  |  |  | 11.88 | 4.97 |
| Hou 2019 | 32 | 20.34 | 1.47 | 0 | 55.84 | 4.01 |
| Jeon 2022 | 355 |  |  | 39.2 | 1.65 | 0.54 |
| Karaibrahimoglu 2023 | 356 |  |  | 84.8 | 48.8 | 15.7 |
| King 2013: PUI only | 76 |  |  |  | 46.4 | 11.7 |
| King 2013: PVG and PUI | 36 |  |  |  | 45.5 | 9.1 |
| King 2013: PVG only | 23 |  |  |  | 44.5 | 12.3 |
| Lin 2024: emotional loneliness* | 1652 |  |  |  | 5.2 | 1.31 |
| Lin 2024: social loneliness* | 1652 |  |  |  | 5.01 | 1.47 |
| Liu 2023 | 23 |  |  |  | 45.73 | 8.54 |
| Myrseth 2017 | 49 |  |  |  | 5.87 | 4.39 |
| Orsolini 2023 | 1261 |  |  | 68.9 | 53.5 | 11.8 |
| Paschke 2022 | 45 | 13.53 | 0.3 | 33.33 | 15.53 | 4.16 |
| Reed 2015 | 192 | 37.25 | 16.18 | 53.64 |  |  |
| Sangram 2020 | 30 |  |  | 7.5 | 47.4 | 6.27 |
| Shettar 2017 | 26 |  |  |  | 55.85 | 5.63 |
| Shi 2017 | 679 |  |  |  | 2.226 | 0.79 |
| Smith 2022 | 15 | 21.6 | 2.2 | 100 | 6.87 | 2 |
| Van Rooij 2014: boys | 174 |  |  | 0 | 1.97 | 0.73 |
| Van Rooij 2014: girls | 30 |  |  | 100 | 2.06 | 0.71 |
| Verma 2023 | 262 |  |  | 58.78 | 14.09 | 4.72 |
| Wang 2022 | 4892 |  |  | 68.2 |  |  |
| Yu 2022 | 337 |  |  |  | 2.2 | 1 |
| Zakaria 2023 | 320 |  |  | 71.5 |  |  |

Table S10 Description of samples of individuals at risk of internet-use disorders

N is the sample size of the group or groups at risk of internet-use disorders or mild symptoms within a study

|  | **n** | **Mean age in years** | **Sd age** | **Sex (% female)** | **Loneliness** | **Sd loneliness** |
| --- | --- | --- | --- | --- | --- | --- |
| Erol 2019 | 89 |  |  |  | 43.06 | 9.77 |
| Hardie 2007: emotional loneliness | 50 |  |  |  | 12.84 | 5.5 |
| Hardie 2007: social loneliness | 50 |  |  |  | 11.88 | 4.04 |
| Liu 2023 | 55 |  |  |  | 43.9 | 7.32 |
| Sangram 2020 | 114 |  |  | 27 | 40.2 | 6.86 |
| Shettar 2017 | 33 |  |  |  | 53.03 | 5.51 |

Table S11 Description of samples of individuals without internet-use disorders

N is the sample size of the group or groups without internet-use disorders within a study, sd is standard deviation. * indicates that the two lines share the same subjects.

| **ID** | **n** | **Mean age in years** | **Sd age** | **Sex (% female)** | **Mean loneliness** | **Sd loneliness** |
| --- | --- | --- | --- | --- | --- | --- |
| Akbari 2023 | 1,766 |  |  |  | 5.8 | 2.1 |
| Aktepe 2013 | 1,408 |  |  | 43.25 | 22.29 | 7.31 |
| Erol 2019 | 384 |  |  |  | 37.83 | 9.56 |
| Hardie 2007 | 38 |  |  |  | 9.71 | 4.73 |
| Hardie 2007 | 38 |  |  |  | 10.34 | 3.83 |
| Hou 2019 | 32 | 20.45 | 1.34 | 0 | 51.8 | 4.56 |
| Jeon 2022 | 327 |  |  | 56.6 | 1.57 | 0.43 |
| Karaibrahimoglu 2023 | 356 |  |  | 84.8 | 44.8 | 15.2 |
| King 2013 | 1,026 |  |  |  | 40.7 | 9.7 |
| Lin 2024: emotional loneliness* | 1,652 |  |  |  | 4.7 | 1.22 |
| Lin 2024: social loneliness* | 1,652 |  |  |  | 4.69 | 1.43 |
| Liu 2023 | 143 |  |  |  | 39.02 | 8.54 |
| Myrseth 2017: non gamers | 164 |  |  |  | 4.03 | 3.14 |
| Myrseth 2017: non-problem gamers | 804 |  |  |  | 3.95 | 3.36 |
| Orsolini 2023 | 1,261 |  |  | 68.9 | 45.6 | 12.3 |
| Paschke 2022 | 914 | 13.55 | 0.07 | 47.59 | 11.34 | 4.23 |
| Reed 2015 | 313 |  |  |  |  |  |
| Sangram 2020 | 56 |  |  |  | 32.16 | 3.9 |
| Shettar 2017 | 41 |  |  |  | 51.66 | 6.95 |
| Shi 2017 | 2,610 |  |  |  | 1.889 | 0.675 |
| Smith 2022 | 158 | 23.8 | 6.1 | 64.3 | 5.2 | 2.4 |
| Van Rooij 2014: boys | 3,434 |  |  | 0 | 1.61 | 0.48 |
| Van Rooij 2014: girls | 2,355 |  |  | 100 | 1.58 | 0.51 |
| Verma 2023 | 262 |  |  | 58.78 | 11.84 | 4.37 |
| Wang 2022 | 2,542 |  |  |  |  |  |
| Yu 2022 | 2,236 |  |  |  | 1.8 | 0.8 |
| Zakaria 2023 | 320 |  |  | 71.5 |  |  |

Table S12 Loneliness as a categorical variable

| **ID** | **N with internet-use disorder symptoms and loneliness** | **N with internet-use disorder symptoms (No loneliness)** | **N with loneliness (no internet-use disorders)** | **N without loneliness or internet-use disorders** | **Odds ratio** | **Lower bound of the confidence interval** | **Upper bound of the confidence interval** |
| --- | --- | --- | --- | --- | --- | --- | --- |
| Reed 2015 | 46 | 146 | 32 | 281 | 2.77 | 1.69 | 4.54 |
| Zhao 2018 | 14 | 27 | 100 | 452 | 2.34 | 1.19 | 4.63 |
| Wang 2022: moderate loneliness |  |  |  |  | 3.36 | 3.08 | 3.66 |
| Zakaria 2023 | 78 | 82 | 83 | 237 | 2.72 | 1.83 | 4.05 |
| Wang 2022: severe loneliness |  |  |  |  | 10.68 | 9.38 | 12.16 |

Table S13 Final outcomes and effect sizes

NPG: non-problematic behavior, DG: disordered gaming, PG: problematic gambling, PUSM: Problematic use of social media, AD: addiction, GD: gambling disorder, C: controls, AR: at risk of addiction, PUI: problematic use of the internet, PVG: problematic video gaming. LBCI: Lower bound of the confidence interval, UBCI: Upper bound of the confidence interval. When multiple samples from the same study are described, we have included additional details of the comparison (following author´s descriptions) to differentiate them.

| **ID** | **Additional details of the comparison** | **Cohen´s d** | **LBCI** | **UBCI** | **Loneliness in those with internet-use disorder symptoms** | **Sd loneliness in those with internet-use disorder symptoms** | **Loneliness in those without internet-use disorders** | **Sd loneliness in those without internet-use disorders** |
| --- | --- | --- | --- | --- | --- | --- | --- | --- |
| Akbari 2023 | NPB vs PG+PUSM&GD+DG&PUSM | 0.528 | 0.435 | 0.62 | 6.91 | 2.12 | 5.80 | 2.10 |
| Aktepe 2013 |  | -0.277 | -0.415 | -0.139 | 20.25 | 7.65 | 22.29 | 7.31 |
| Erol 2019 | AD vs C+AR | 0.002 | -0.496 | 0.5 | 38.80 | 8.78 | 38.78 | 9.60 |
| Erol 2019 | C vs AD+AR | 0.466 | 0.248 | 0.683 | 42.28 | 9.60 | 37.83 | 9.56 |
| Hardie 2007 | AD vs C+AR (emotional and social loneliness combined) | 0.256 | -0.256 | 0.769 |  |  |  |  |
| Hardie 2007 | C vs AD+AR | 0.492 | 0.198 | 0.785 |  |  |  |  |
| Hou 2019 |  | 0.941 | 0.424 | 1.457 | 55.84 | 4.01 | 51.80 | 4.56 |
| Jeon 2022 |  | 0.163 | 0.013 | 0.314 | 1.65 | 0.54 | 1.57 | 0.43 |
| King 2013 | No PVG or PUI vs PUI+VPG+PVG&PUI | 0.519 | 0.342 | 0.695 | 45.80 | 10.87 | 40.70 | 9.70 |
| King 2013 | No PVG or PUI + PUI only vs PVG only+PVG and PUI | 0.434 | 0.172 | 0.697 | 45.24 | 10.03 | 40.98 | 9.81 |
| King 2013 | No PVG or PUI + PVG only vs PUI only+PVG and PUI | 0.534 | 0.338 | 0.73 | 46.01 | 10.64 | 40.75 | 9.74 |
| Lin 2024 | Emotional and social loneliness combined | 0.314 | 0.135 | 0.49 |  |  |  |  |
| Liu 2023 | AD vs C+AR | 0.615 | 0.18 | 1.05 | 45.73 | 8.54 | 40.70 | 8.14 |
| Liu 2023 | C vs AD+AR | 0.645 | 0.363 | 0.93 | 44.33 | 7.62 | 39.02 | 8.54 |
| Myrseth 2017 | Non gaming + Non problem gaming vs problem gaming | 0.564 | 0.276 | 0.852 | 5.87 | 4.39 | 3.97 | 3.32 |
| Orsolini 2023 |  | 0.648 | 0.532 | 0.77 | 53.50 | 11.80 | 45.60 | 12.3 |
| Paschke 2022 |  | 0.991 | 0.688 | 1.293 | 15.53 | 4.16 | 11.34 | 4.23 |
| Reed 2015 |  | 0.244 | -0.21 | 0.698 | 20.93 | 13.97 |  |  |
| Sangram 2020 | AD vs C+AR | 2.217 | 1.772 | 2.662 | 47.40 | 6.27 | 35.35 | 5.28 |
| Sangram 2020 | C vs AD+AR | 1.608 | 1.262 | 1.955 | 41.93 | 6.72 | 32.16 | 3.90 |
| Shettar 2017 | AD vs C+AR | 0.566 | 0.112 | 1.02 | 55.85 | 5.63 | 52.43 | 6.18 |
| Shettar 2017 | C vs AD+AR | 0.419 | 0.016 | 0.822 | 54.24 | 5.56 | 51.66 | 6.95 |
| Shi 2017 |  | 0.481 | 0.396 | 0.566 | 2.23 | 0.79 | 1.89 | 0.68 |
| Smith 2022 |  | 0.705 | 0.17 | 1.239 | 6.87 | 2.00 | 5.20 | 2.40 |
| Van Rooij 2014 | Boys | 0.727 | 0.574 | 0.881 | 1.97 | 0.73 | 1.61 | 0.48 |
| Van Rooij 2014 | Girls | 0.936 | 0.575 | 1.297 | 2.06 | 0.71 | 1.58 | 0.51 |
| Verma 2023 |  | 0.501 | 0.293 | 0.71 | 14.09 | 4.72 | 11.84 | 4.37 |
| Wang 2022 | Moderate+Severe loneliness | 0.375 | 0.309 | 0.441 |  |  |  |  |
| Yu 2022 |  | 0.483 | 0.367 | 0.598 | 2.20 | 1.00 | 1.80 | 0.80 |
| Zakaria 2023 |  | 0.239 | -0.127 | 0.61 |  |  |  |  |

Table S14 Risk of bias evaluation (NOS)

|  | **SELECTION** |  |  |  | **COMPARABILITY** | **OUTCOME** | **TOTAL** |
| --- | --- | --- | --- | --- | --- | --- | --- |
|  | **Representativeness** | **Size** | **Non-respondents** | **Ascertainment** | **Comparability** | **Outcome** |  |
| Akbari 2023 | 0 | 0 | 0 | 0 | 0 | 1 | 1 |
| Aktepe 2013 | 1 | 1 | 0 | 0 | 0 | 1 | 3 |
| Erol 2019 | 1 | 0 | 0 | 0 | 0 | 1 | 2 |
| Hardie 2007 | 0 | 0 | 0 | 0 | 0 | 1 | 1 |
| Hou 2019 | 0 | 0 | 0 | 0 | 0 | 1 | 1 |
| Jeon 2022 | 1 | 1 | 1 | 0 | 0 | 1 | 4 |
| Karaibrahimoglu 2023 | 1 | 1 | 0 | 0 | 0 | 1 | 3 |
| King 2013 | 1 | 0 | 0 | 0 | 0 | 1 | 2 |
| Lin 2024 | 1 | 1 | 1 | 0 | 0 | 1 | 4 |
| Liu 2023 | 0 | 0 | 1 | 0 | 0 | 1 | 2 |
| Myrseth 2017 | 1 | 0 | 0 | 0 | 0 | 1 | 2 |
| Orsolini 2023 | 0 | 1 | 0 | 0 | 0 | 1 | 2 |
| Paschke 2022 | 1 | 1 | 1 | 0 | 0 | 1 | 4 |
| Reed 2015 | 0 | 0 | 0 | 0 | 0 | 1 | 1 |
| Sangram 2020 | 0 | 0 | 0 | 0 | 0 | 1 | 1 |
| Shettar 2017 | 1 | 0 | 0 | 0 | 0 | 1 | 2 |
| Shi 2017 | 1 | 0 | 0 | 0 | 0 | 1 | 2 |
| Smith 2022 | 1 | 0 | 0 | 0 | 0 | 1 | 2 |
| Van Rooij 2014 | 1 | 1 | 1 | 0 | 0 | 1 | 4 |
| Verma 2023 | 1 | 0 | 1 | 0 | 0 | 1 | 3 |
| Wang 2022 | 1 | 1 | 1 | 0 | 0 | 1 | 4 |
| Yu 2022 | 1 | 0 | 1 | 0 | 0 | 1 | 3 |
| Zakaria 2023 | 0 | 0 | 0 | 0 | 0 | 1 | 1 |

Table S15 Meta-regression analyses

Coeff: Coefficient, Std err: standard error, Z: Z-value, P>|z|: p-value associated to the Z-value. LBCI: Lower bound of the confidence interval, UBCI: Upper bound of the confidence interval.

^a^ Values are compared to Western countries (Australia, Germany, Italy, Netherlands, Norway, UK, k=8). East Asia included the following countries: China, India, Korea, Malaysia, and Taiwan, k=11. Africa and West Asia included Iran and Turkey; k=4. The study from Trinidad and Tobago was left out of this analysis due to being the sole study from the Caribbean region.

|  | Values for variable of interest | | | | | | Values for constant | | | | | |
| --- | --- | --- | --- | --- | --- | --- | --- | --- | --- | --- | --- | --- |
| Variable | Coeff. | Std  err | z | P>\|z\| | LBCI | UBCI | Coeff. | Std err | z | P>\|z\| | LBCI | UBCI |
| Mean age | -.0110454 | .0178662 | -0.62 | 0.536 | -.0460625 | .0239718 | .7577202 | .3630013 | 2.09 | 0.037 | .0462508 | 1.46919 |
| % females overall | -.0005017 | .004095 | -0.12 | 0.902 | -.0085277 | .0075243 | .5536382 | .2281094 | 2.43 | 0.015 | .1065519 | 1.000725 |
| Overall sample size | -.0005525 | .0004849 | -1.14 | 0.255 | -.001503 | .0003979 | .6420105 | .131296 | 4.89 | 0.000 | .384675 | .8993459 |
| Country^a^ |  |  |  |  |  |  | .6254153 | .1472035 | 4.25 | 0.000 | .3369018 | .9139288 |
| East Asia | -.0371283 | .193077 | -.19 | 0.848 | -.4155522 | .3412957 |  |  |  |  |  |  |
| Africa and West Asia | -.4866149 | .2511791 | -1.94 | 0.053 | -.9789169 | .005687 |  |  |  |  |  |  |

# *Supplementary Figures*

Figure S1 Main analysis, funnel plot

*
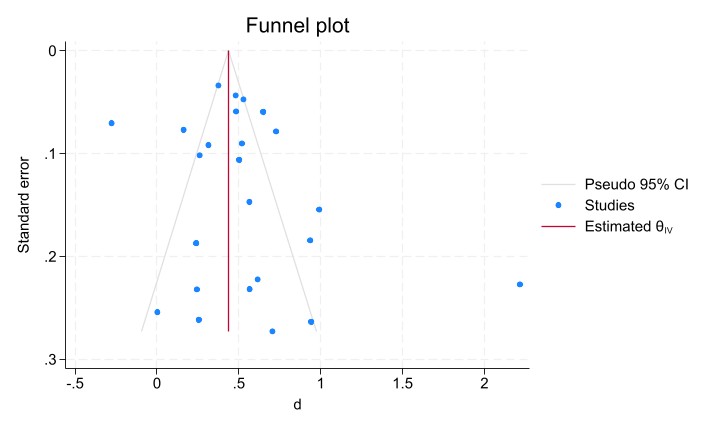
*

Figure S2 Main analysis, leave-one-out forest plot

*
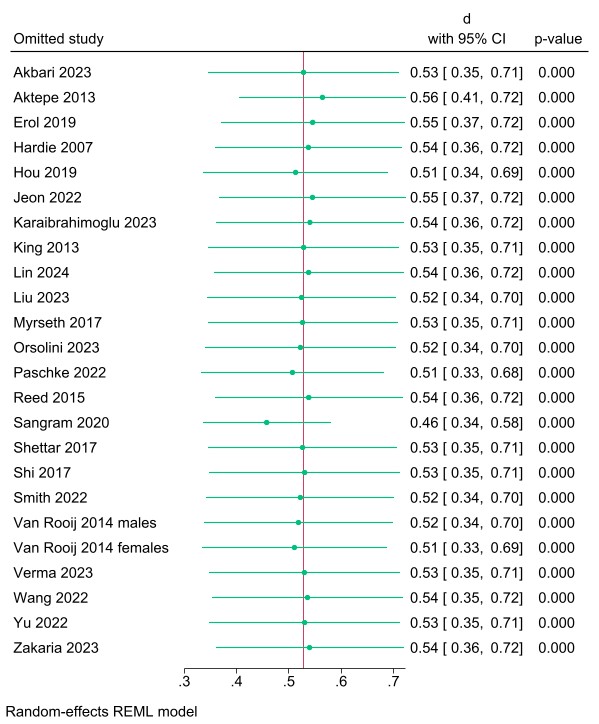
*

Figure S3 Analysis permitting a lower threshold for internet-use disorder symptoms

*
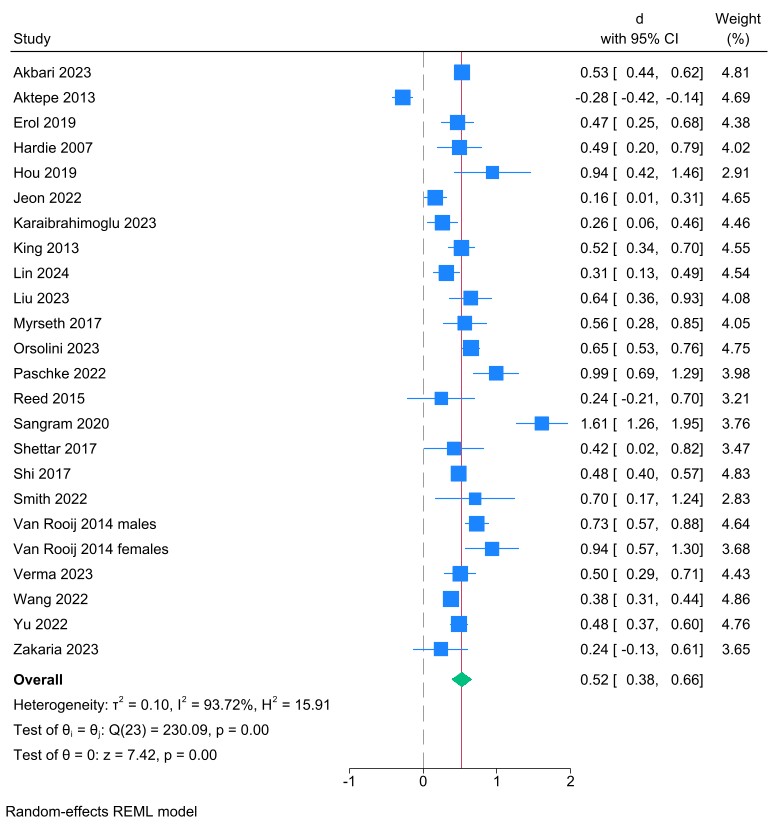
*

Figure S4 Pooled average in the UCLA-LS for individuals without internet-use disorders

*
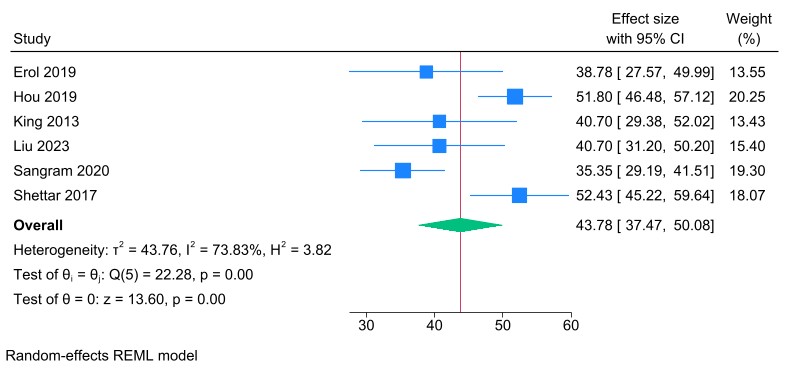
*

Figure S5 Pooled average in the UCLA-LS for individuals with internet-use disorder symptoms

*
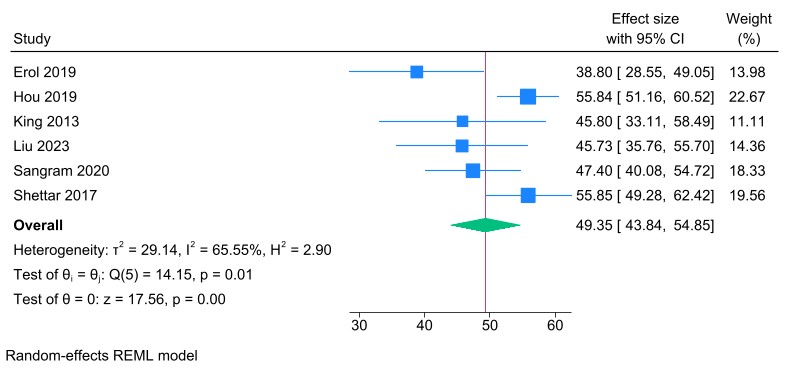
*

Figure S6 Standardized mean difference between groups in the UCLA-LS

*
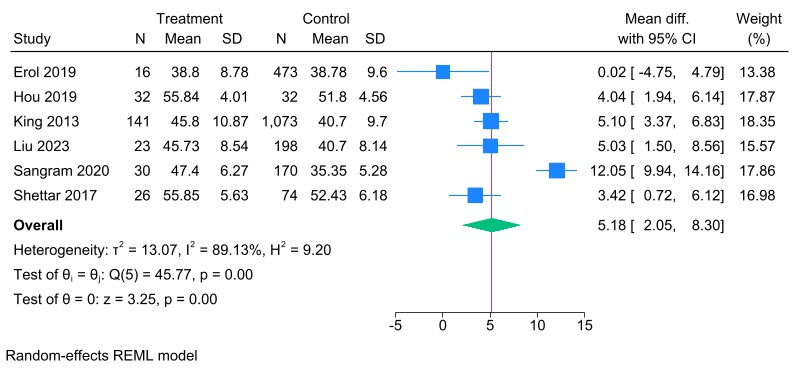
*
